# Alprazolam exposure during adolescence induces long-lasting dysregulation in reward sensitivity to morphine and second messenger signaling in the VTA-NAc pathway

**DOI:** 10.1038/s41598-023-37696-8

**Published:** 2023-07-05

**Authors:** Astrid M. Cardona-Acosta, Omar K. Sial, Lyonna F. Parise, Tamara Gnecco, Giselle Enriquez Marti, Carlos A. Bolaños-Guzmán

**Affiliations:** 1 Department of Psychological and Brain Sciences and Program in Neuroscience, Texas A&M University, College Station, TX 77843 USA; 2 Department of Neuroscience, The Scripps Research Institute, Jupiter, FL USA; 3 Fishberg Department of Neuroscience, Friedman Brain Institute, Icahn School of Medicine at Mount Sinai, New York, NY USA

**Keywords:** Reward, Behavioural methods, Motivation

## Abstract

Increased use of benzodiazepines in adolescents have been reported, with alprazolam (ALP) being the most abused. Drug abuse during adolescence can induce changes with lasting consequences. This study investigated the neurobiological consequences of ALP exposure during adolescence in C57BL/6J male mice. Mice received ALP (0, 0.5, 1.0 mg/kg) once/daily (postnatal day 35–49). Changes in responsiveness to morphine (2.5, 5.0 mg/kg), using the conditioned place preference paradigm, were assessed 24-h and 1-month after ALP exposure. In a separate experiment, mice received ALP (0, 0.5 mg/kg) and then sacrificed 24-h or 1-month after treatment to assess levels of extracellular signal regulated kinase 1/2 (ERK1/2) gene expression, protein phosphorylation, and downstream targets (CREB, AKT) within the ventral tegmental area (VTA) and nucleus accumbens (NAc). ALP-pretreated mice developed a strong preference to the compartment(s) paired with a subthreshold dose (2.5 mg/kg) of MOR short-term, and this effect was also present in the 1-month group. Adolescent ALP exposure resulted in dysregulation of ERK-signaling within the VTA-NAc pathway 24-h and 1-month after ALP exposure. Results indicate ALP exposure during adolescence potentiates the rewarding properties of MOR and induces persistent changes in ERK-signaling within the VTA-NAc pathway, a brain circuit highly implicated in the regulation of both drug reward and mood- related behaviors.

## Introduction

Benzodiazepines (BDZs) are widely prescribed for the treatment of insomnia, convulsive and anxiety-related disorders. However, they possess adverse side effects such as amnesia, tolerance, dependence, and high potential for addiction. Concerning trends in BDZ abuse have been consistently reported in the past few decades implicating BDZs in approximately one-third of unintentional drug overdoses^1,2^. In addition, BDZ abuse often occurs in conjunction with other substances (e.g., alcohol, opioids) with about 33% of opioid overdose deaths in the U.S. involving BDZ co-ingestion^3^. Alprazolam (Xanax; ALP) is a highly potent and short-acting BDZ that is among the most prescribed psychotropic medications in the U.S.—of the approximately 92 million BDZ prescriptions dispensed in outpatient pharmacies in 2019, ALP was the most commonly (38%) prescribed^4^. Its high prescription rates have persisted throughout the years despite exerting adverse effects as many other BDZs. While research on the consequences of BDZ use has largely focused on the elderly and their co-prescribing with opioids, much less is known about their use and misuse in the adolescent population. About one in four teens have reported misused/abused prescription medications at least once in their lifetime, and, surprisingly, 20% of these had done so before the age of 14^5^. Despite safety concerns surrounding BDZ treatment utilization, which have prompted guidelines to confine their use as short-term treatment^6^, long-term use of BDZs is rather common, including in pediatric settings^7–9^. This is cause for concern because BDZ prescription trends positively correlate with its nonmedical use among adolescents^10^. To exacerbate this, multiple drug co-ingestion is particularly prevalent in the adolescent population. An estimated 5.3% of 12th graders report engaging in past-year non-medical BDZ use and 72.6% of these users engaged in polydrug use. Furthermore, those engaged in polydrug use were more likely to get “high” from use, to have nonmedically used ALP, and to initiate its use prior to 10th grade^11^. Though it is estimated that only 15% of drug users transition from recreational use to substance use disorder (SUD), the chances of developing SUD increase dramatically when the onset of drug use occurs at a younger age^12–14^, thus emphasizing the need to monitor BDZ use/abuse in this population.

Although they may initially act on different targets, drugs of abuse ultimately strongly activate the same reward circuitry as natural rewards (e.g., food, sex, social interaction): the mesolimbic dopamine (DA) circuit. The reward circuit, partly comprised of the ventral tegmental area (VTA) and one of its output structures, the nucleus accumbens (NAc), is well known to regulate natural reward and mood-related behaviors and is a major target for drugs of abuse. A well-established feature of all abused drugs is that they increase DA levels within this VTA-NAc network and can induce synaptic plasticity changes that aid in the development of addiction^15,16^. It has been proposed that under basal conditions, DA neurons receive local inhibitory inputs from GABA interneurons resulting in the inhibition to other output structures. In the presence of BDZs, GABA interneurons are inhibited and no longer control (i.e., inhibit) DA neurons in the VTA (a process known as disinhibition), which results in increased activity of VTA’s DAergic neurons. The increased activity in VTA DA neurons results in more DA being released in target regions such as the NAc, thus contributing to drug reward^17^. Within the VTA, GABA_A_ and mu (μ)-opioid receptors are thought to be co-localized on inhibitory GABA interneurons^18^. It is therefore feasible that BDZ exposure induces synaptic alterations within the VTA and subsequently on its target regions. These findings have led to the disinhibition hypothesis, which states that BDZs and opioids exert their rewarding effects via disinhibition of GABAergic interneurons within the VTA, thus leading to an increase in DA release into the NAc, a major substrate for motivated behavior. Such molecular mechanism is thought to contribute to the enhancement of reward observed in the co- administration of BDZs and opioids. Nonetheless, it remains unclear how precisely BDZs influence DA release. While electrophysiology measures show that BDZs disinhibit VTA DA neurons^19,20^ microdialysis studies have reported decreases in DA concentrations in the NAc after acute and repeated BDZ administration^21–24^. In addition, fast scan cyclic voltammetry (FSCV) suggests that BDZs decrease the amplitude of electrically evoked accumbal DA concentrations^25^. Recently, it was demonstrated that diazepam increases the frequency of DA release events while also decreasing their amplitude, suggesting that these two effects are a result of different mechanisms^26^. It has been speculated that the discrepancy between electrophysiological and biochemical results may be due to the differential effects of BDZs on the activity of DA neurons in anesthetized animals (electrophysiological experiments) and freely moving animals (in vivo microdialysis), as electrophysiological activity at the level of the cell body of DA neurons may not reflect activity at the terminal (for review see^27,28^). More recently, it was found that activation of local NAc GABA_A_ receptors by diazepam suppresses DA release and that this suppression requires GABA_B_ receptor activation, as application of a GABA_B_ receptor antagonist blocked this effect. These findings suggest that BDZs uniquely influence DA activity: their administration results in opposing effects at the level of cell bodies in the VTA (increase in DA firing) and the terminal region of the NAc to suppress the amount of DA release^29^. Historically, BDZs alone have been shown to be weak reinforcers^30^. Interestingly, clinical reports indicate that opiate users often self-administer BDZs prior to, or concurrently with their opiates to potentiate their rewarding effects^31,32^. Likewise, these effects have been observed in adult rodent models where a single administration of ALP enhanced the rewarding properties of a low dose of heroin that by itself was not rewarding as measured by the conditioned place preference (CPP) paradigm^33^. In a subsequent study, Walker et al.^34^ found that ALP pretreatment enhanced the rewarding effects of intra-VTA heroin induced CPP, thus suggesting that the VTA might be a site where opiate + BDZ interaction occurs. Whether ALP contributes to the enhancement of opioid reward directly via the VTA or indirectly via other mechanism(s) (i.e., local inhibition of NAc GABAergic interneurons) is a question that remains to be elucidated.

Despite being one of the most misused BDZs during adolescence, basic research on the functional consequences of ALP exposure during this critical developmental period is severely lacking. Therefore, this study was designed to investigate the behavioral and neurobiological consequences after ALP exposure in adolescent (postnatal day [PD] 35-49) male mice. Given the prevalence of ALP misuse in adolescence and their co-ingestion with opioids, we assessed the short- and long-term consequences of repeated ALP on behavioral sensitivity to morphine as measured by the CPP paradigm. Because drugs targeting the GABAergic system induce molecular adaptations in the mesolimbic system^35^, we also measured ALP-induced changes in the expression of the extracellular regulated protein kinase 1/2 (ERK1/2) and its downstream target cAMP response element-binding protein (CREB) within the VTA and the NAc, neural substrates implicated in drug reward and mood regulation^36,37^. In addition, we assessed changes in the expression of protein kinase B (AKT) due to its role as molecular regulator of drug reward as seen after repeated opiate administration^38^. Here, we expand on previous work on the modulatory effects of ALP on opioid reward in an adolescent model that has not been studied before. ALP use/abuse is of interest given the mounting evidence of its consumption during this critical period of development and its interaction with the endogenous opioid system.

## Materials and methods

### Animals

C57BL/6J male adolescent mice [postnatal day 28 (PD28) on arrival] were used in this study (Jackson Laboratory; Bar Harbor, ME). Mice were housed (5 per cage) in clear polypropylene boxes containing wood shavings, located in a temperature-controlled (23–25 °C) vivarium maintained on a 12-h light-dark cycle in which the lights were on between 7:00 A.M. and 7:00 P.M. Food and water were provided ad libitum throughout the course of the experiments. All experimental procedures were performed in strict accordance with the Guidelines for the Care and Use of Mammals in Neuroscience and Behavioral Research (National Research Council 2003), the ARRIVE Guidelines, and approved by the Texas A&M University Animal Care and Use committee.

### Drugs

Alprazolam (ALP), a benzodiazepine (BDZ), was purchased from Spectrum Pharmaceuticals (Irvine, CA). Due to its insolubility in water, the vehicle (VEH) solution consisted of 9:1 ratio 0.9% sterile saline and kolliphor. ALP was administered in a volume of 4 ml/kg intraperitoneal (i.p.). Morphine (MOR) sulfate was obtained from Spectrum Pharmaceuticals (Irvine, CA) and was dissolved in 0.9% sterile saline. Morphine was delivered subcutaneously (s.c.) in a volume of 4 ml/kg.

### Drug treatment

Mice were randomly assigned to the various experimental conditions: drug (VEH or ALP) and time of behavioral/biochemical assessment (short-term: 24 h, or long-term: 1 month after the last drug exposure). Mice received i.p. injections of VEH or ALP (0.5 and 1.0 mg/kg) once daily for 14 consecutive days (PD35-49). The doses chosen for ALP pretreatment were selected to mimic recreational use based on previous reports showing these doses enhance the liking in human subjects and induce behavioral effects in animal models^30,39,40^. The drug pretreatment period between PD35-49 was chosen as it roughly parallels adolescence in humans^41,42^. Mice assigned to the short-term behavioral and biochemical conditions were tested 24 h after the last injection (PD50), while those assigned to the long-term condition were left undisturbed and tested 1 month after the last injection (PD79), a point in which they had reached adulthood. For the CPP experiments, subthreshold doses of MOR (2.5, 5.0 mg/kg), which do not induce place conditioning on their own, were selected to determine whether ALP pre-exposure would influence behavioral effects in response to MOR.

### Conditioned place preference (CPP)

Conditioned place preference (CPP) to MOR was performed in a three-compartment apparatus where each compartment differed in wall coloring and floor texture. On the preconditioning day (day 0), mice were allowed to explore the entire apparatus for 30 min to obtain baseline preference to any of the three compartments (length by width by height: side compartments, 35 × 27 × 25 cm; middle compartment, 10 × 27 × 25 cm). Mice did not show any preference for either side compartment (before MOR exposure). Conditioning trials occurred over three consecutive days. During conditioning days 1–3 the mice received a saline (SAL) injection in the morning and were confined to one of the side compartments of the apparatus for 1 h. After an intermission period of 4 h, mice received MOR (0, 2.5, 5.0 mg/kg, s.c.) in the afternoon and were confined to the opposite side compartment of the apparatus (drug-paired compartment) for 1 h. On the test day (day 4), the mice were allowed to explore the entire apparatus for 30 min under a drug-free state and time spent in the drug-paired compartment was assessed. The test was performed in the middle of the day to control the mice from making potential associations with VEH or drug injection based on time of day. Place conditioning was calculated as total time spent in the MOR-paired compartment minus total time spent in the SAL-paired compartment on test day.

### RNA extraction and quantitative real-time PCR

Mice were sacrificed 24 h (short-term) and 1 month (long-term) after repeated ALP exposure. Brains were extracted and sliced into 1-mm diameter coronal sections. A 14-gauge needle was used to collect VTA and NAc punches that were rapidly stored at − 80 °C until assayed. RNA was isolated using RNeasy Micro Kit (Qiagen) according to manufacturer’s instructions and cDNA was then created from these samples using the Applied Biosystems High-Capacity cDNA Reverse Transcription Kit (Thermo-Fisher). Quantitative real-time PCRs were performed in triplicates using 384 well PCR plates and RealMasterMix (Eppendorf) with Eppendorf MasterCycler Realplex2 according to the manufacturer’s instructions. Threshold cycle [C(t)] values were measured using the supplied software and analyzed using the ΔΔC(t) method as described previously^43,44^. Primer sequences for ERK1 (Mapk3), ERK2 (Mapk1), CREB (creb1), AKT (Akt), and glyceraldehyde-3-phosphate dehydrogenase (Gapdh) are listed on Table 1. [*Mapk3:* Mitogen activated protein kinase 2 (ERK1); *Mapk1*: Mitogen activated protein kinase 1 (ERK2); *Creb1*: cAMP response element binding protein 1 (CREB); *Akt:* Protein kinase B (AKT); *Gapdh:* glyceraldehyde-3-phosphate dehydrogenase (GAPDH)].

**Table 1:** qPCR primers.

Gene	Primer Sequence	
	Forward	Reverse
*Mapk3*	5’-GTACATCGGAGAAGGCGCCTAC-3’	5’-TTGTAAAGGTCCGTCTCCAT-3’
*Mapk1*	5’-GGTTGTTCCCAAATGCTGACT-3’	5’-CAACTTCAATCCTCTTGTGAGGG-3’
*Creb1*	5’-AGTGACTGAGGAGCTTGTACCA-3’	5’-TGTGGCTGGGCTTGAAC-3’
*Akt*	5’-GCACCTTTATTGGCTACAAGGA-3’	5’-GGGGACTCTCGCTGATCCA-3’
*Gapdh*	5’-AGGTCGGTGTGAACGGATTTG-3’	5’-TGTAGACCATGTAGTTGAGGTCA-3’

### Western blotting

In a separate cohort, mice received repeated ALP exposure and were sacrificed 24 h and 1 month after the last injection for western blot analysis. Protein from VTA and NAc tissue punches were sonicated in standard lysis buffer and then centrifuged at 14,000 rpm for 15 min. Ten micrograms of protein from each sample were treated with β-mercaptoethanol and electrophoresed on precast 4–20% gradient gels (Bio-Rad), as described previously^45^^,^^46^. All antibodies were obtained from Cell Signaling (Beverly, Massachusetts). Blots were probed overnight at a 4 °C with antibodies against the phosphorylated forms of ERK1/2, CREB, AKT and GAPDH. Membranes were stripped with Restore Pierce Biotechnology (Rockford, Illinois) and re-probed with antibodies against the total forms of ERK1/2, CREB, AKT and GAPDH. All primary antibodies were diluted to a 1:1000 concentration. Membranes were washed several times with TBST and were then incubated with peroxidase-labeled goat anti-rabbit IgG (1:10,000; Cell signaling, Beverly, Massachusetts). Bands were visualized with SuperSignal West Dura substrate (Pierce Biotechnology, Rockford, IL), quantified using ImageJ (NIH), and subsequently normalized to GAPDH.

### Statistical analysis

The behavioral data was analyzed using a two-way analysis of variance (ANOVA) with ALP pre- treatment and MOR treatment as sources of variance. Post-hoc comparisons were analyzed using Tukey’s test. When appropriate, Student’s *t* tests were used to determine statistical significance of pre-planned comparisons. Data are expressed as the mean ± SEM. Statistical significance was defined as *p* < 0.05.

## Results

### Short- and long-term effects of repeated ALP administration during adolescence on body weight of C57BL/6J mice

**Figure 1:**
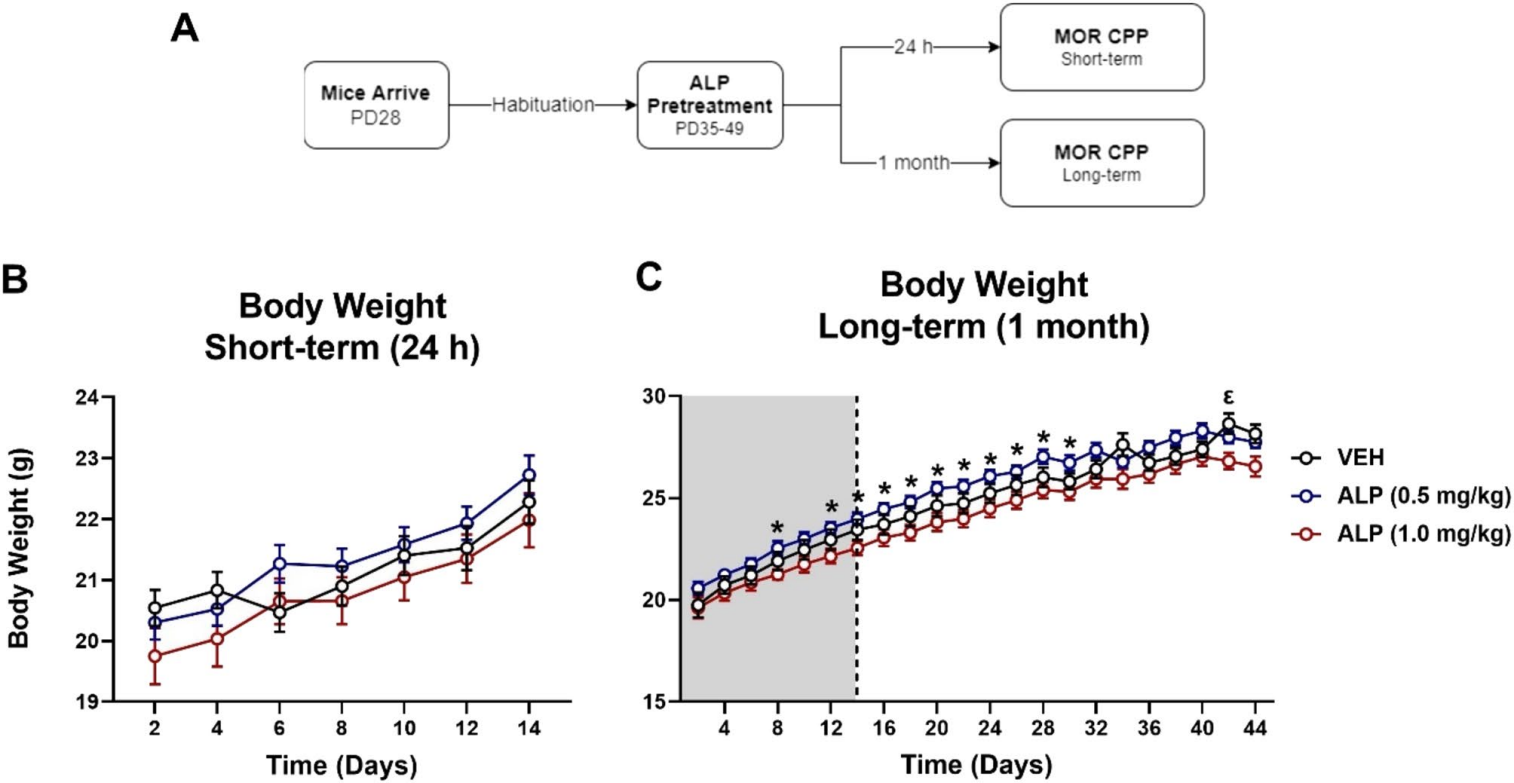
Short- and long-term effects of adolescent repeated ALP exposure on body weight. Body weights of *C57BL/6 J* male adolescent mice throughout repeated alprazolam exposure. (**A**) Mice were habituated for one week (PD28-34) then exposed to 14 days to either vehicle (VEH) or alprazolam (ALP; n = 12/group). The mice were exposed to the conditioned place preference (CPP) paradigm with morphine (MOR) either 24 h (Short-term) or 1 month (Long-term) after the last ALP injection. (**B**) Mice in the short-term condition gained weight over time (*p* < 0.0001) regardless of treatment condition. (**C**) Likewise, mice in the long-term condition gained weight over time (*p* < 0.0001), with the ALP 0.5 mg/kg weighting more than the ALP 1.0 mg/kg group (*p* < 0.05). Shaded region indicates days of ALP exposure. *p < 0.05 when compared to ALP (1.0 mg/kg) pretreated mice. ^ε^*p* < 0.05 when compared to VEH-pretreated mice. All data are expressed as the mean ± SEM.

Body weight was measured every other day throughout ALP pretreatment in both short- and long- term groups and continued to be measured 1 month after cessation of treatment in the long-term group (Fig. 1A–C). A two-way repeated measures ANOVA showed that mice in the short-term condition gained weight over time (*F*(2,76) = 138.1, *p* < 0.001) but did not differ from each other as a function of pretreatment exposure (*F*(2,33) = 0.777, *p* = 0.4679; Fig. 1B). In addition, there was an interaction of time and drug pretreatment (*F*(12,198) = 4.091, *p* < 0.001). In the long-term group, a mixed-effects analysis showed that all mice gained weight over time (*F*(3,91) = 540.6, *p* < 0.0001), and this varied as a function of drug pretreatment (*F*(2,33) = 3.359, *p* < 0.05). Tukey’s post hoc comparisons revealed that mice pretreated with 0.5 mg/kg ALP were heavier than those pretreated with 1.0 mg/kg ALP on day 8 and days 12–30 (*p* < 0.05, respectively). Furthermore, VEH-pretreated mice were heavier than those pretreated with 1.0 mg/kg ALP on day 42 (*p* < 0.05). Interestingly, upon further analysis of the long-term group, we found no differences in body weight between experimental groups during the 14-days of ALP pretreatment (*p* < 0.001).

### Short- and long-term effects of repeated ALP administration during adolescence on morphine place conditioning

**Figure 2:**
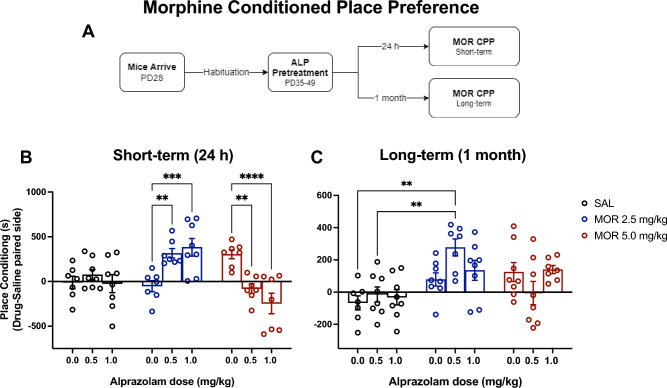
Short- and long-term effects of adolescent repeated ALP exposure on morphine place conditioning. Effects of repeated exposure to vehicle (VEH) or alprazolam (ALP) during adolescence on morphine-induced conditioned place preference. (**A**) Mice were habituated for one week (PD28-34) then exposed to 14 days to either vehicle (VEH) or alprazolam (ALP; n = 7–8/group). (**B**) Short-term (24 h after the last injection): mice pretreated with 0.5 and 1.0 mg/kg ALP showed an increased preference for the subthreshold dose of morphine (MOR, 2.5 mg/kg) when compared to VEH-pretreated controls. Conversely, mice pretreated with 1.0 mg/kg ALP showed aversion-like behavior to a subthreshold dose of MOR (5.0 mg/kg) when compared to the VEH- pretreated mice. (**C**) Long-term (1 month after the last injection): mice pretreated with 0.5 mg/kg ALP showed an increased preference for the subthreshold dose of MOR (2.5 mg/kg) when compared to VEH-pretreated controls and SAL-conditioned mice. *p < 0.05, **p < 0.01, ***p < 0.001, ****p < 0.0001.

To test for changes in behavioral responsiveness to MOR reward, place preference conditioning was assessed either 24 h (short-term; n = 7–8/group) or 1 month (long-term; n = 7–8/group) following repeated exposure to VEH or ALP during adolescence (Fig. 2A–C). As expected, VEH-pretreated mice did not show place conditioning to the subthreshold doses of MOR (2.5, 5.0 mg/kg) when compared to the SAL treated controls (*p* > 0.05), regardless of treatment and time conditions. In the short-term group, time spent in the MOR-paired compartment was not influenced by ALP pretreatment, but varied as a function of MOR treatment (*F*(2,57) = 7.428, *p* < 0.0014) and by an interaction between the two variables (*F*(4,57) = 10.74, *p* < 0.0001). Mice pretreated with ALP, regardless of dose, readily conditioned to the compartments paired with a subthreshold dose of MOR (2.5 mg/kg) when compared with the VEH-pretreated mice (*p* < 0.01 and *p* < 0.001, respectively; Fig. 2B). Interestingly, mice pretreated with ALP, regardless of dose, spent more time in the SAL-paired compartment, significantly avoiding the compartments paired with the 5.0 mg/kg MOR, when compared to VEH-pretreated mice (*p* < 0.01 and *p* < 0.0001, respectively). The magnitude of the MOR-induced place conditioning showed by the ALP-pretreated (0.5 or 1.0 mg/kg) mice was not significantly different for each MOR treatment (*p* > 0.05). No differences were found between 0.5 mg/kg ALP pretreated mice when compared to SAL-treated controls for each MOR dose (*p* > 0.05, respectively). In addition, significant differences in place conditioning were observed between 1.0 mg/kg ALP at the 2.5 (*p* < 0.01), but not at the 5.0 MOR dose (*p* > 0.05) when compared to SAL-treated controls.

When assessing long-term effects (Fig. 2C), time spent in the drug-paired compartments was significantly influenced by MOR treatment (*F*(2,60) = 11.21, *p* < 0.0001), and varied as a function of the interaction between the variables (*F*(4,60) = 2.925, *p* = 0.0282). Mice pretreated with 0.5 mg/kg ALP showed a preference for the compartments paired with 2.5 mg/kg MOR when compared to the 0.5 mg/kg ALP + SAL (*p* < 0.001) and the VEH + SAL treated mice (*p* < 0.001). No differences were found between the 0.5 and 1.0 mg/kg ALP-pretreated mice when compared to VEH-pretreated controls regardless of MOR dose (*p* > 0.05).

### Short-term effects of repeated ALP administration during adolescence on ERK-related signaling within the VTA

**Figure 3:**
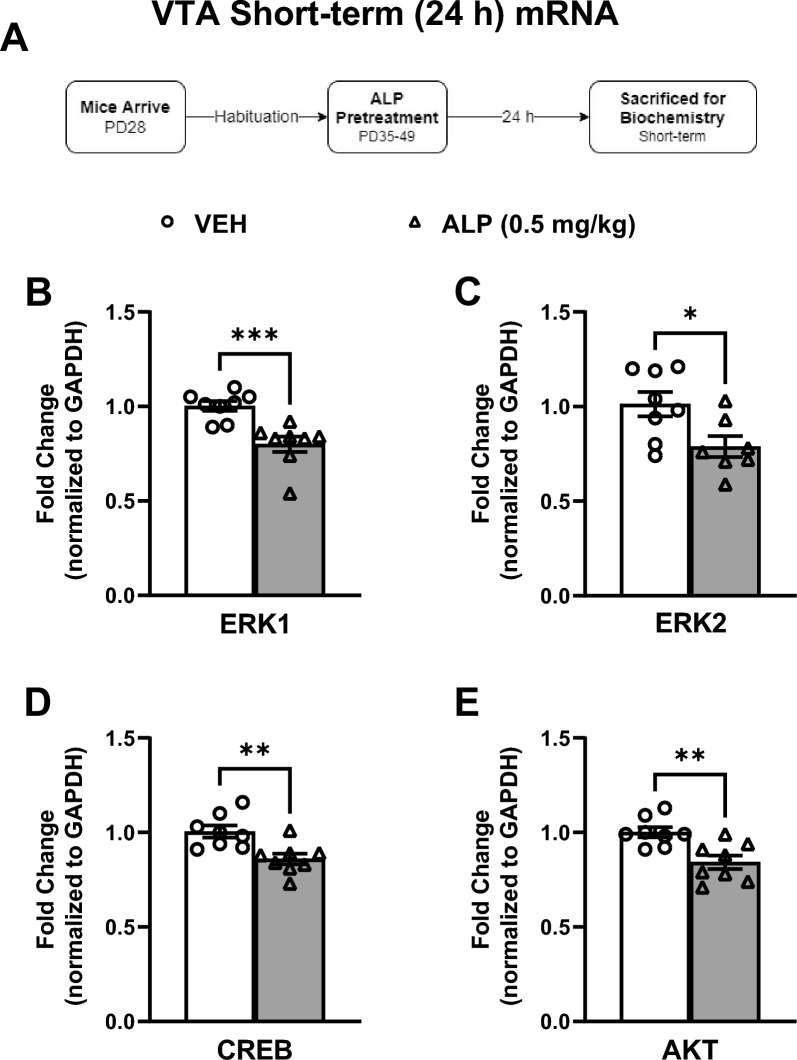
Short-term effects of adolescent repeated ALP exposure on ERK-related gene expression within the VTA 24 h after the last injection. (**A**) Effects of repeated exposure to vehicle (VEH) or alprazolam (ALP; 0.5 mg/kg) in adolescent male mice on ERK-related gene expression within the ventral tegmental (VTA) 24 h after the last injection (short-term). (**B**) ERK1 (*p* < 0.001); (**C**) ERK2 (*p* < 0.05); (**D**) CREB (*p* < 0.01); and (**E**) AKT (*p* < 0.01) mRNA levels were significantly decreased by ALP when compared to VEH-treated mice. Data are represented as fold change normalized to GAPDH (mean ± SEM).

**Figure 4:**
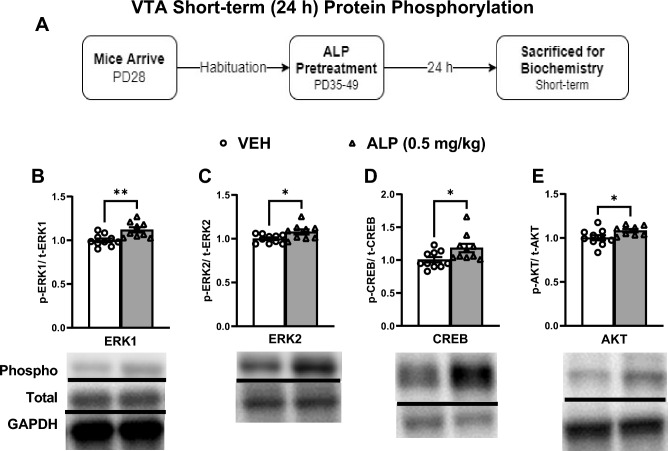
Short-term effects of adolescent repeated ALP exposure on ERK-protein phosphorylation within the VTA 24 h after the last injection. (**A**) Effects of repeated vehicle (VEH) or alprazolam (ALP; 0.5 mg/kg) exposure in adolescent male mice on protein phosphorylation within the ventral tegmental area (VTA) 24 h after the last injection. Treatment with ALP significantly increased (**B**) ERK1, (**C**) ERK2, (**D**) CREB, and (**E**) AKT phosphorylated forms when compared to VEH-treated mice (*p* < 0.05, respectively).*p < 0.05, **p < 0.01 when compared to VEH-treated controls. Data are presented as a ratio of total protein normalized to GAPDH (mean ± SEM). See supplementary materials for full-length blots.

Gene expression was assessed within the VTA 24 h after repeated VEH or 0.5 mg/kg ALP exposure in adolescent mice (Fig. 3A–E; n = 8–10/group). ALP treatment significantly decreased ERK1 (*t*(14) = 4.175, *p* < 0.001; Fig. 3B), ERK2 (*t*(13) = 2.594, *p* < 0.05; Fig. 3C), CREB (*t*(14) = 3.428, *p* < 0.001; Fig. 3D), and AKT (*t*(14) = 3.549, *p* < 0.01; Fig. 3E) mRNA expression when compared to the VEH-treated mice. The activity of ERK-related signaling within the VTA was further assessed as inferred from the phosphorylation of ERK protein and its downstream targets (Fig. 4A–E; n = 8–10/group; all normalized to GAPDH and presented as a ratio of phosphorylated form over total protein expression). ALP pretreatment significantly increased the phosphorylation of ERK1 (*t*(17) = 3.349, *p* < 0.01; Fig. 4B), ERK2 (*t*(17) = 2.407, *p* < 0.001; Fig. 4C), CREB (*t*(18) = 2.357, *p* < 0.05; Fig. 4D) and AKT (*t*(16) = 2.158, *p* < 0.05; Fig. 4E) protein when compared to the VEH-treated controls. No change in total ERK1, ERK2, CREB, AKT, or GAPDH protein levels were detected when compared to VEH-treated controls (*p* > 0.05, see supplementary materials).

### Short-term effects of repeated ALP administration during adolescence on ERK-related signaling within the NAc

**Figure 5:**
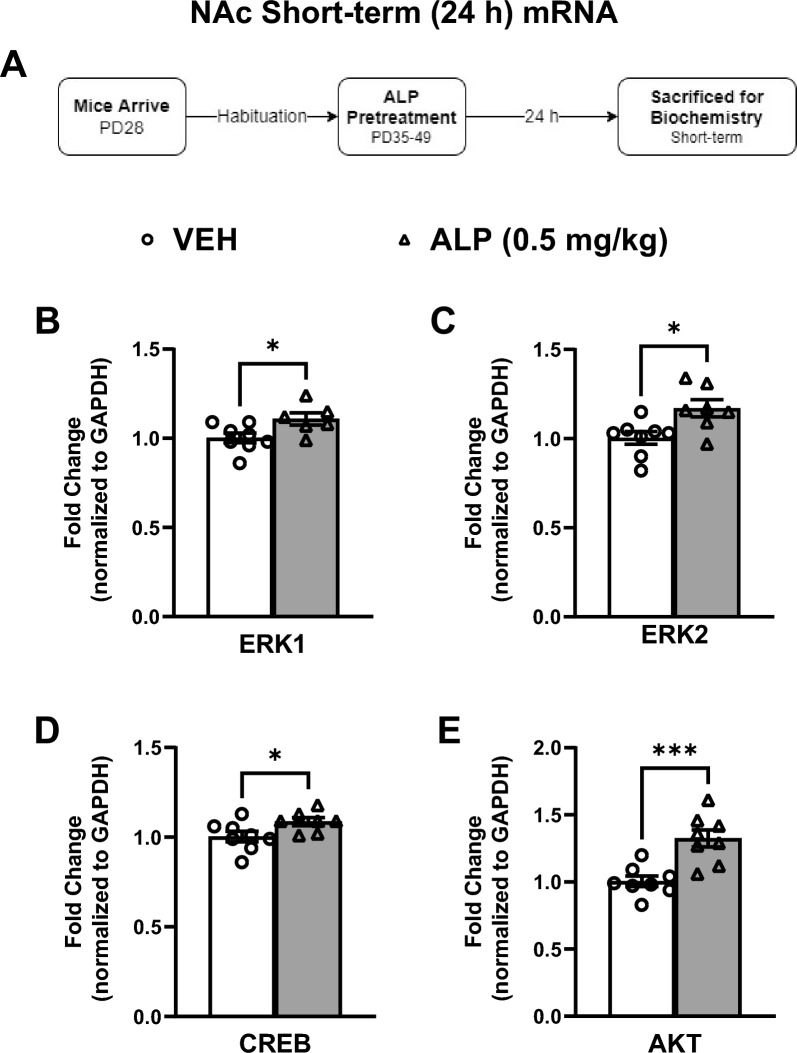
Short-term effects of adolescent repeated ALP exposure on ERK-related gene expression within the NAc 24 h after the last injection. (**A**) Effects of repeated vehicle (VEH) or alprazolam (ALP; 0.5 mg/kg) exposure in adolescent male mice on ERK-related gene expression within the nucleus accumbens (NAc) 24 h after the last injection. (**B**) ERK1 (*p* < 0.05); (**C**) ERK2 (*p* < 0.05); (**D**) CREB (*p* < 0.05); and (**E**) AKT (*p* < 0.001) mRNA levels were significantly increased by ALP when compared to VEH-treated controls. *p < 0.05, ***p < 0.001 compared to VEH-treated controls. Data are represented as fold change normalized to GAPDH (mean ± SEM).

**Figure 6:**
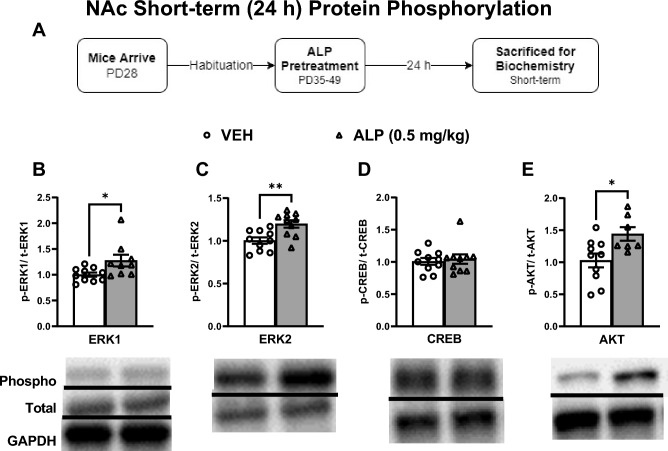
Short-term effects of adolescent repeated ALP exposure on ERK-protein phosphorylation within the NAc 24 h after the last injection. (**A**) Effects of repeated vehicle (VEH) or alprazolam (ALP; 0.5 mg/kg) exposure in adolescent male mice on protein phosphorylation within the nucleus accumbens (NAc) 24 h after the last injection. Treatment with ALP significantly increased (**B**) ERK1, (**C**) ERK2, and (**E**) AKT phosphorylated forms when compared to VEH-treated controls (*p* < 0.05, respectively). There was no change in (**D**) CREB phosphorylation when compared to VEH-treated controls. *p < 0.05, **p < 0.01 when compared to VEH-treated controls. Data are presented as a ratio of total protein normalized to GAPDH (mean ± SEM). See supplementary materials for full-length blots.

Gene expression was also assessed within the NAc 24 h after repeated VEH or 0.5 mg/kg ALP exposure in adolescent mice (Fig. 5A–E; n = 8–10/group). Mice treated with ALP showed significant increases in ERK1 (*t*(12) = 2.468, *p* < 0.05; Fig. 5B), ERK2 (*t*(13) = 2.841, *p* < 0.05; Fig. 5C), CREB (*t*(13)=2.239, *p*<0.05; Fig. 5D) and AKT (*t*(14)=4.287, *p*<0.001; Fig. 5E) mRNA expression when compared to the VEH-treated mice. Likewise, the assessment of ERK-related protein phosphorylation was performed within the NAc after repeated ALP treatment (Fig. 6A–E; n=8–10/group; all normalized to GAPDH and presented as a ratio of phosphorylated form to total protein expression). ALP treatment significantly increased the phosphorylated forms of ERK1 (*t*(17)=2.363, *p*<0.05; Fig. 6B), ERK2 (*t*(18)=3.344, *p*<0.01; Fig. 6C), and AKT (*t*(15)=2.646, *p*< 0.05; Fig. 6E), while no changes were observed in CREB (*t*(18)=0.4005, *p*=0.693; Fig. 6D) when compared to the VEH-treated controls. No changes were observed in total ERK1, ERK2, CREB, AKT or GAPDH (*p*>0.05, see supplementary materials).

### Long-term effects of repeated ALP administration during adolescence on ERK-related signaling within the VTA

**Figure 7:**
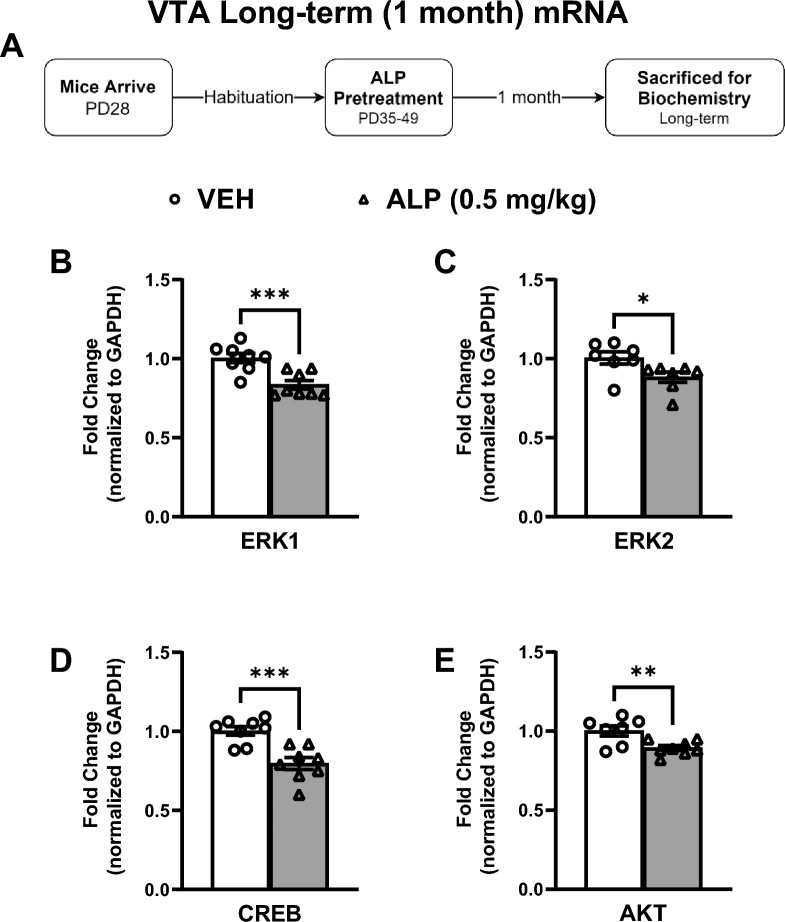
Long-term effects of adolescent repeated ALP exposure on ERK-related gene expression within the VTA 1 month after the last injection. (**A**) Effects of repeated vehicle (VEH) or alprazolam (ALP; 0.5 mg/kg) exposure in adolescent male mice on ERK-related gene expression within the ventral tegmental area (VTA) 1 month after the last injection. (**B**) ERK1 (*p* < 0.001), (**C**) ERK2 (*p* < 0.05), (**D**) CREB (*p* < 0.001), and (**E**) AKT (*p* < 0.01) mRNA levels were significantly decreased by ALP when compared to VEH-treated controls. *p < 0.05, **p < 0.01, ***p < 0.001 compared to VEH-treated controls. Data are represented as fold change normalized to GAPDH (mean ± SEM).

**Figure 8:**
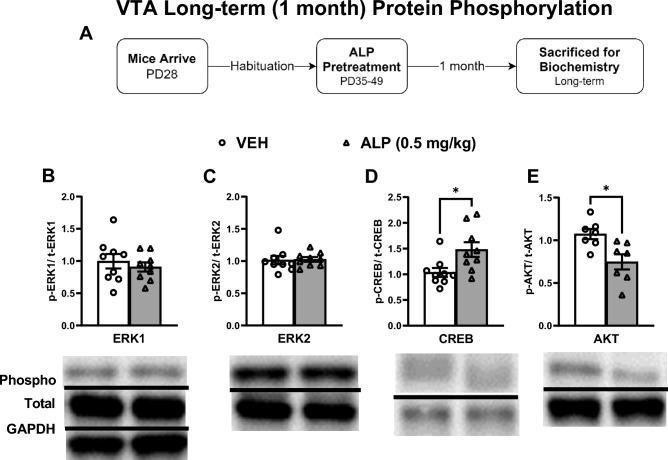
Long-term effects of adolescent repeated ALP exposure on ERK-protein phosphorylation within the VTA 1 month after the last injection. (**A**) Effects of repeated vehicle (VEH) or alprazolam (ALP; 0.5 mg/kg) exposure in adolescent male mice on protein phosphorylation within the ventral tegmental area (VTA) 1 month after the last injection. Treatment with ALP had no effects on (**B**) ERK1, and (**C**) ERK2 phosphorylation when compared to VEH-treated controls. ALP significantly increased (**D**) CREB phosphorylation when compared to VEH-treated controls. ALP treatment significantly decreased (**E**) AKT phosphorylation when compared to VEH-treated controls. *p < 0.05 when compared to VEH-treated controls. Data are presented as a ratio of total protein normalized to GAPDH (mean ± SEM). See supplementary materials for full-length blots.

To test for potential long-lasting effects, gene expression within the VTA was assessed 1 month after the last exposure to VEH or 0.05 mg/kg ALP (Fig. 7A–E; n = 8–10/group). ALP treatment induced significant decreases in ERK1 (*t*(14) = 4.155, *p* < 0.001; Fig. 7B), ERK2 (*t*(12) = 2.430, *p* < 0.05; Fig. 7C), CREB (*t*(14) = 4.416, *p* < 0.001; Fig. 7D), and AKT (*t*(13) = 3.129, *p* < 0.01; Fig. 7E) mRNA expression when compared to the VEH-treated mice. We further assessed ERK-related protein phosphorylation within the VTA 1 month after the cessation of repeated ALP treatment (Fig. 8A–E; n = 8–10/group; all normalized to GAPDH and presented as a ratio of phosphorylated form to total protein expression). Interestingly, ALP treatment did not affect ERK1 (*t*(16) = 0.6722, *p *= 0.5111; Fig. 8B) or ERK2 (*t*(16) = 0.1486, *p*=0.8837; Fig. 8C) phosphorylated forms when compared to the VEH-treated mice. However, ALP exposure during adolescence increased levels of CREB (*t*(16) = 2.648, *p*<0.05; Fig. 8D) and decreased levels of AKT (*t*(12) = 3.035, *p* < 0.05; Fig. 8E) protein phosphorylation when compared to their respective VEH-treated controls. No changes in total levels of ERK1, ERK2, CREB, AKT, or GAPDH were detected when compared to the VEH- treated controls (*p* > 0.05, see supplementary materials).

### Long-term effects of repeated ALP administration during adolescence on ERK-related signaling within the NAc

**Figure 9:**
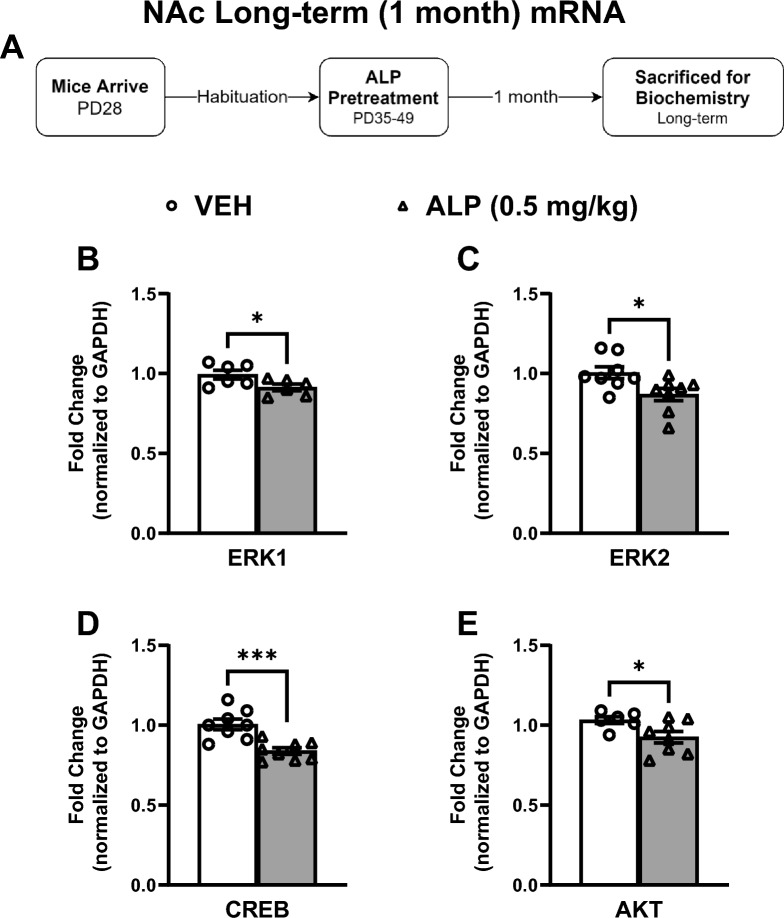
Long-term effects of adolescent repeated ALP exposure on ERK-related gene expression within the NAc 1 month after the last injection. (**A**) Effects of repeated vehicle (VEH) or alprazolam (ALP; 0.5 mg/kg) exposure in adolescent male mice on ERK-related gene expression within the nucleus accumbens (NAc) 1 month after the last injection. ALP exposure significantly decreased (**B**) ERK1 (*p* < 0.05); (**C**) ERK2 (*p* < 0.05); (**D**) CREB (*p* < 0.001); and (**E**) AKT (*p* < 0.05) mRNA levels when compared to VEH-treated controls. *p < 0.05, ***p < 0.001 compared to VEH-treated controls. Data are represented as fold change normalized to GAPDH (mean ± SEM).

**Figure 10:**
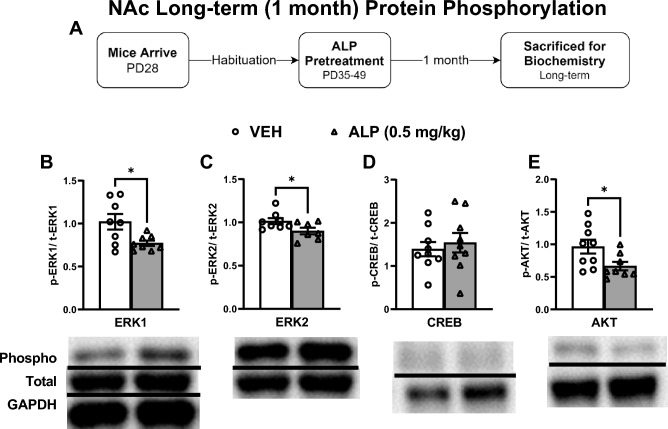
Long-term effects of adolescent repeated ALP exposure on ERK-protein phosphorylation within the NAc 1 month after the last injection. (**A**) Effects of repeated vehicle (VEH) or alprazolam (ALP; 0.5 mg/kg) exposure in adolescent male mice on protein phosphorylation within the nucleus accumbens (NAc) 1 month after the last injection. ALP treatment significantly decreased (**B**) ERK1, (**C**) ERK2, and (**E**) AKT phosphorylated forms when compared to VEH-treated controls (*p* < 0.05, respectively). Treatment with ALP induced no effects on (**D**) CREB phosphorylation when compared to VEH-treated controls. *p < 0.05 when compared to VEH-treated controls. Data are presented as a ratio of total protein normalized to GAPDH (mean ± SEM). See supplementary materials for full-length blots.

We also measured the effects of adolescent exposure to VEH or 0.5 mg/kg ALP on gene expression within the NAc 1 month after cessation of treatment to assess for potential long-term effects (Fig. 9A–E; n = 8–10/group). ALP treatment induced significant decreases in ERK1 (*t*(10) = 2.307, *p* < 0.05; Fig. 9B), ERK2 (*t*(14) = 2.574, *p* < 0.05; Fig. 9C), CREB (*t*(14) = 4.321, *p* < 0.001; Fig. 9D), and AKT (*t*(12) = 2.329, *p* < 0.05; Fig. 9E) mRNA expression when compared to the VEH-pretreated mice. ERK-related protein phosphorylation was also assessed within this brain region (Fig. 10A–E; n = 8–10/group; all normalized to GAPDH and presented as ratio of phosphorylated form to total protein expression). ALP treatment induced significant decreases in ERK1 (*t*(14) = 2.624, *p* < 0.05; Fig. 10B), ERK2 (*t*(13) = 2.199, *p* < 0.05; Fig. 10C), and AKT (*t*(15) = 2.342, *p* < 0.05; Fig. 10E) phosphorylated forms, while having no effect on CREB (*t*(16) = 0.5259, *p* = 0.6062; Fig. 10D) when compared to the VEH-treated controls. No changes in total levels of ERK1, ERK2, CREB, AKT or GAPDH were detected when compared to VEH-treated controls (*p* > 0.05, see supplementary materials).

## Discussion

This study assessed the short- (24-h) and long-term (1-month after the las injection) neurobiological consequences of alprazolam (ALP) exposure during adolescence (PD35-49), a drug that is both highly prescribed^47^ and abused by the adolescent population in the U.S.^48^, yet little is known about the functional consequences of ALP exposure during this critical developmental period. We report that repeated ALP during adolescence in male mice results in changes in body weight, enhancement of behavioral reactivity to morphine (MOR), as measured in the conditioned place preference (CPP) paradigm, and dysregulation of the extracellular signal-regulated kinase (ERK1/2) and related downstream signaling within the VTA and NAc, brain regions highly implicated in regulation of drug reward and mood-related behavior^36,37,49^. Surprisingly, the behavioral effects of ALP pretreatment on MOR-induced CPP lasted into adulthood.

Repeated ALP resulted in changes in body weight in the long-term group, with no effects observed during ALP pretreatment. BDZs have been shown to influence the ingestion of food and drink by enhancing taste palatability^50^. Thus, changes in body weight could be due, at least in part, to ALP’s influence of taste palatability. However, it remains unclear whether these effects are dose-dependent^51,52^^.^ Previous work suggests that BDZ-induced enhancement in taste palatability is not due to changes in sensory characteristics of food but in the central positive hedonic evaluation of the taste and food. However, the hyperphagic response varies depending on the drug’s actions at the GABA_A_ receptor (full vs. partial agonists) and dose^50^. In addition, some BDZs do not exert hyperphagic effects, making it difficult to determine the extent to which BDZs enhances sensitivity to natural rewards^53^.

We assessed the short- and long- term effects of repeated ALP exposure on behavioral responses to morphine as measured by the CPP paradigm. Twenty-four hours after the last injection, adolescent mice pretreated with ALP (0.5, 1.0 mg/kg) showed a preference for environments previously paired with 2.5 mg/kg MOR, a subthreshold dose that had no significant effects in the VEH-pretreated mice. Surprisingly, the mice avoided the compartment paired with the 5.0 mg/kg MOR dose when compared to the VEH-pretreated controls (i.e., an aversion-like behavior profile). This suggests a leftward shift in the dose response curve for MOR after ALP, as ALP-pretreated mice showed a significant behavioral reactivity to both subthreshold doses of MOR (place reference at 2.5; place avoidance at 5.0 mg/kg) that by themselves had no effects on CPP in their respective controls. These results can be explained within the framework of MOR’s biphasic effects (i.e., inverted U-shape dose response curve) where higher doses of MOR can become aversive^54^. Our results are in concert with previous work showing that ALP-pretreatment modulates opioid drug reward in adult rodents^53,54^. Interestingly, there were no differences in the magnitude of the 2.5 mg/kg MOR-induced place conditioning developed by mice treated with the different ALP doses (0.5 and 1.0 mg/kg), suggesting that ALP similarly influenced the system to induce sensitivity to the low MOR dose. No differences were found between 2.5 mg/kg MOR-treated groups when compared to SAL-treated controls for each of ALP pretreatments (0.5, 1.0 mg/kg). The mechanism(s) underlying the avoidance behavioral profile observed is unknown, as the subthreshold doses of MOR (2.5, 5.0 mg/kg) by themselves are not known to induce place preference or aversion, thus a possibility is that pretreatment with ALP is modulating the behavior observed^18,34,35^. The effects of repeated BDZ administration on opioid receptor regulation have not been fully elucidated. A recent study showed that buprenorphine (a partial μ-opioid receptor agonist) promotes rapid desensitization and downregulation of receptors, resulting in a reduction in agonist efficacy. However, ALP administration prior to buprenorphine exposure restores the density of μ-opioid receptors binding sites. Indeed, ALP was classified as one of the most active BDZs in µ-opioid receptor regulation^55^. It is therefore plausible that ALP exposure prior to MOR upregulates the density of μ-opioid receptors which, in turn, may enhance the binding of MOR once it is introduced, and therefore intensifying its pharmacological effects. Given that MOR possess biphasic effects^56^, it is thus possible that ALP preexposure increased receptor number and/or affinity such that when coupled with a moderate dose of MOR may induce aversion-like behavior effects.

The long-term effects of ALP exposure during adolescence on MOR CPP were also assessed. Mice pretreated with 0.5 mg/kg ALP showed preference for the environment paired with low dose of MOR (2.5 mg/kg) when compared to SAL-treated controls 1 month after cessation of pretreatment, suggesting that repeated ALP induces changes that last into adulthood. No differences were found between the ALP-pretreated mice when compared to VEH-pretreated controls for MOR regardless of dose. As expected, MOR did not influence place conditioning in the VEH-pretreated when compared to SAL-treated controls. The chronic use of BDZs is known to induce tolerance, physical dependence, and withdrawal during periods of abstinence^57^. The development of physical dependence is dependent on timing and rate of exposure, dose, and drug potency. A longer timeframe of use, higher doses, and higher drug potency, increase the likelihood of dependence^58–61^. In humans, doses vary from 0.25 to 0.5 mg for up to three times a day leads to tolerance and dependence^62^. In animal models, physical dependence has been reported at doses ≥ 1 mg/kg administered twice a day^63,64^. In the current study, the ALP doses (0.5, 1.0 mg/kg; one injection/day) were selected to mimic recreational use based on work showing these doses enhance the drug liking in human subjects and induce behavioral effects in animal models^39^. The doses used in this study are relatively low where the development of physical dependence is questionable. However, tolerance to BDZs can develop in the absence of physical dependence^57^. In addition, dysregulation of mood and reward pathways can occur after repeated administration even at low doses^65,66^. Although speculative given that the present study does not have an adult comparison group, the enhanced response to MOR observed in ALP-pretreated mice may be age specific. Adolescence is a developmental period marked by enhanced sensitivity to drugs of abuse, and vulnerability stemming from the significant restructuring that occurs in the brain^41,67^. Indeed, DA neurons have been shown to fire faster in adolescent rodents, potentially because GABA tone increases as animals reach adulthood^68^, and this elevation in firing rate is consistent with increased addiction liability during adolescence^68^. Our results are consistent with the literature indicating that exposure to drugs of abuse early in life leads to long-lasting neural alterations resulting in behavioral changes that last into adulthood^69,70^.

Given the behavioral findings indicating that ALP pretreatment influences behavioral responsiveness to subthreshold doses of MOR, the 0.5 mg/kg ALP dose was chosen in subsequent experiments to assess the molecular consequences of its repeated exposure (14 days) during adolescence. We measured the expression of ERK-related signaling within the VTA and NAc 24 h and 1 month after the last ALP exposure. These brain regions were selected since it has been hypothesized that BDZs exert their rewarding properties by interaction with the VTA and NAc, a neural circuit that is a major substrate for motivated behavior, responses to natural rewards and drugs of abuse^71,72^. Intracellular pathways such as ERK and IRS2-AKT are highly regulated by stress and drugs of abuse and involved in regulation of mood-related disorders and drug-induced neuroplasticity^73,74^. To this end, we measured the levels of gene expression of ERK1/2 and its downstream molecular targets. Twenty-four hours from the last ALP injection, we observed significant decreases in ERK1/2, CREB, and AKT mRNA within the VTA. The functional significance of these ALP-induced changes is unknown. Because an increase/decrease in gene expression does not always correlate with similar results in protein levels^75^, short-term protein phosphorylation levels were also assessed. Increases in ERK1, ERK2, CREB and AKT phosphorylation were found within the VTA while total levels of protein expression remained unchanged. The discrepancy between these findings might be due to post-translational modifications (i.e., acetylation, hydroxylation, ubiquitination) that may change the functional state, catalytic activity, or signaling of these kinases^76^. Moreover, differences in neurochemical profile (GABAergic, dopaminergic, glutamatergic neurons) of the VTA may add yet another layer of complexity. It is also possible that the changes observed are VTA-region-specific, which the technique used to harvest the tissue does not allow for more precise determination.

Changes in gene expression within the NAc were also assessed 24 h after repeated ALP exposure. ALP induced increases in ERK1, ERK2, CREB, and AKT mRNA. Although novel within the framework of adolescent drug exposure, these results were not surprising as a well- established neurobiological response to repeated administration of addictive drugs is the increase in DA levels within the NAc where the encoding of incentive-motivational valence of drugs is hypothesized to occur^77^. Repeated ALP exposure increased ERK1, ERK2, and AKT phosphorylation within the NAc, while total protein levels remained unchanged, thus confirming the activity of these enzymes. Previous studies have shown that ERK plays a critical role in the development of sensitization to drugs of abuse, and its blockade within the NAc inhibits the expression of sensitization^78^. Alterations in glutamate signaling and plasticity within the VTA and NAc also play a critical role in the expression of psychomotor sensitization of stimulants ^79,80^. Similarly, long-lasting modulation of glutamatergic transmission in VTA DA neurons have been observed after BDZ treatment, including the increase in ratio of AMPA/NMDA receptors^71^. Although speculative, the increases in ERK phosphorylation within the VTA and NAc lend support for the notion that repeated ALP treatment may induce increased sensitivity to the behavioral effects of MOR (a sensitization-like state) such that rewarding stimuli produces a greater increase in neurotransmission within these brain regions. Within the context of drug use and abuse, a neural system that is sensitized or “hypersensitive” is hypothesized to mediate psychosocial functions such as the increase in incentive salience of stimuli that may lead to the “wanting” of the drug^81^. Our results support the notion that repeated ALP treatment dysregulates sensitivity to the behavioral effects of MOR, as ERK is known to induce molecular adaptations that increase sensitivity to drugs of abuse within these brain regions^82^. Given the complexity of the VTA’s neurochemical profile, it is possible that ALP influences reward-related behaviors by directly acting on the NAc. GABA_A_ receptors exist on medium spiny neurons (MSN) in the NAc, thus it is possible that modulation of DA sensitivity is enhanced by direct stimulation of GABA_A_ receptors on MSN^83^. Chronic treatment with GABA_A_ receptor agonist zolpidem enhanced sensitization to morphine-induced hyperlocomotion and enhanced mesolimbic dopaminergic activity through the up-regulation of post-synaptic KCC2, a transmembrane co-transporter. KCC2 blockade in the NAc inhibited morphine-induced hyperlocomotion^84^. These findings contradict the evidence that BDZs reduce DA levels in the NAc, however it is likely that BDZs influence abuse potential to other drugs of abuse through both direct and indirect mechanisms within the VTA-NAc circuit. Nonetheless, changes induced by chronic treatment with ALP during adolescence may influence the vulnerability to opioid abuse via molecular changes within this pathway.

Repeated ALP administration during adolescence also induced long-lasting dysregulation of ERK-signaling within the VTA-Nac pathway. Changes in ERK signaling were assessed 1 month after cessation of ALP treatment. Within the VTA, we observed decreases in ERK1, ERK2, CREB and AKT mRNA levels. Surprisingly, there were no effects in ERK1/2 protein phosphorylation within this brain region. The lack of significant effects may be due to cell-specific kinetics parameters, such as the availability of free ribosomes to initiate translation^75,85^. Interestingly, we observed increases in CREB and decreases in AKT phosphorylation. The functional significance of CREB and AKT dysregulation induced by ALP is unknown; however, the activity of these kinases bears a resemblance to what is observed during periods of abstinence from opioids and responses to stressful stimuli^86,87^. Within the NAc, decreases in ERK1/2 mRNA and its downstream targets were observed. This was followed by decreases in ERK1/2 and AKT protein phosphorylation, while no changes in CREB activity were observed. Similarly to findings in the VTA, decreases in phosphorylated ERK in the NAc have been associated with the incubation of heroin seeking that is induced by drug cues during periods of abstinence^88^. These molecular effects suggest that, although to a lesser extent than opioids, ALP exposure/abstinence induce a negative emotional state. It has been well-documented that during periods of BDZ abstinence individuals experience a rebound in anxiety^58,89^. It is therefore possible that repeated ALP exposure primes the system in a way (via negative emotional states) that facilitates further drug seeking/intake when an animal is reintroduced to drug-associated context cues or to the drug itself. Thus, repeated ALP exposure during adolescence may pose long-lasting detrimental effects as it may render the system vulnerable to drug intake/abuse later in life. Taken together, this data supports the notion that repeated administration of drugs of abuse, particularly early in life, may induce dysregulation of second-messenger systems that may result in enduring aberrant behavioral consequences later in life. See Table 2 for a summary of the biochemistry results. [Significantly upregulated ( ↑ ), downregulated ( ↓ ), or no change ( < > ) compared with VEH-treated controls.] Importantly, the current work has considerable relevance for understanding the behavior of drug users who co-abuse BDZs and opioids. It is important to note, however, that the findings reported here are derived from an adolescent model. Studies on the potential enduring effects of ALP exposure during adolescence in humans are lacking, making interpretative parallels challenging. Nevertheless, it is possible to conceive the notion that ALP exposure early in life may influence responsiveness to drugs of abuse and subsequent drug taking behavior in adulthood. The findings presented herein thus necessitate to be expanded to investigate the effects of repeated ALP exposure during adolescence on functional outcomes in adulthood given that SUDs are more likely to emerge when drug exposure starts early in life^13,14^. It is also of great importance to replicate the current work in an adolescent female model given that females may respond differently to drugs of abuse when compared to males^90^. Alarmingly, recent trends indicate that women are prescribed BDZs at higher rates than men, and overdose death among women, particularly involving BDZs, have increased substantially^91,92^. Together, the findings from future work will help us better understand the behavioral and neurobiological effects of BDZs on opioid reward and provide insights into the development of better therapeutics that minimize the risk for developing SUDs/addiction.

**Table 2:** ERK-related signaling after repeated alprazolam (ALP) treatment within the ventral tegmental area (VTA) and nucleus accumbens (NAc) 24 h or 1 month after the last injection.

Treatment		Interval	Brain region	Gene	mRNA ΔΔC(t)	Protein phosphorylation (phospho/total)
Repeated	ALP	24 h	VTA	ERK1	↓	↑
Repeated	ALP	24 h	VTA	ERK2	↓	↑
Repeated	ALP	24 h	VTA	CREB	↓	↑
Repeated	ALP	24 h	VTA	AKT	↓	↑
Repeated	ALP	24 h	NAc	ERK1	↑	↑
Repeated	ALP	24 h	NAc	ERK2	↑	↑
Repeated	ALP	24 h	NAc	CREB	↑	< >
Repeated	ALP	24 h	NAc	AKT	↑	↑
Repeated	ALP	1 month	VTA	ERK1	↓	< >
Repeated	ALP	1 month	VTA	ERK2	↓	< >
Repeated	ALP	1 month	VTA	CREB	↓	↑
Repeated	ALP	1 month	VTA	AKT	↓	↓
Repeated	ALP	1 month	NAc	ERK1	↓	↓
Repeated	ALP	1 month	NAc	ERK2	↓	↓
Repeated	ALP	1 month	NAc	CREB	↓	< >
Repeated	ALP	1 month	NAc	AKT	↓	↓

## Conclusion

Repeated ALP exposure during adolescence increased the rewarding effects of a low dose of MOR, behavioral effects that persisted into adulthood. ALP exposure induced short-term increases in ERK1/2 signaling within the VTA-NAc pathway, and though speculative, repeated ALP may be sensitizing this neural system resulting in an enhancement of opioid reward. One month after ALP cessation, decreases in ERK1/2-related signaling were observed within this pathway, molecular effects resembling those observed during periods of abstinence from opioids, suggesting that ALP exposure poses long-lasting detrimental effects that may facilitate drug intake later in life. See Table 2.

## Supplementary Information

Supplementary Figures.

Supplementary Figures.

## Data Availability

All data will be made available from the corresponding author upon reasonable request.

## Abbreviations

ALP: Alprazolam
MOR: Morphine
BDZ: Benzodiazepine
CPP: Conditioned place preference
ERK: Extracellular signal regulated kinase
VTA: Ventral tegmental area
NAc: Nucleus accumbens
DA: Dopamine
GABA: Gamma-aminobutyric acid
CREB: CAMP response element-binding protein
AKT: Protein kinase B

## References

[1] Henderson A, Wright M, Pond SM (1993). Experience with 732 acute overdose patients admitted to an intensive care unit over six years. Med. J. Aust..

[2] Jones CM, Mack KA, Paulozzi LJ (2013). Pharmaceutical overdose deaths, United States, 2010. JAMA.

[3] Gladden RM, O’Donnell J, Mattson CL, Seth P (2019). Changes in opioid-involved overdose deaths by opioid type and presence of benzodiazepines, cocaine, and methamphetamine—25 states, July-December 2017 to January-June 2018. MMWR Morb. Mortal. Wkly. Rep..

[4] U.S. Food and Drug Administration. FDA requiring boxed warning updated to improved safe use of benzodiazepine drug class (2020). https://www.fda.gov/drugs/drug-safety-and-availability/fda-requiring-boxed-warning-updated-improve-safe-use-benzodiazepine-drug-class.

[5] PATS, 2013 Key Findings: Released July 23, 2014. https://drugfree.org/wp-content/uploads/2014/07/PATS-2013-KEY-FINDINGS1.pdf.

[6] Guina J, Merrill B (2018). Benzodiazepines I: Upping the care on downers: The evidence of risks, benefits and alternatives. J. Clin. Med..

[7] Murphy KD, Sahm LJ, McCarthy S, Byrne S (2015). Benzodiazepine prescribing guideline adherence and misuse potential in Irish minors. Int. J. Clin. Pharm..

[8] Kurko TA (2015). Long-term use of benzodiazepines: Definitions, prevalence and usage patterns—a systematic review of register-based studies. Eur. Psych..

[9] Yeh HH (2011). Five-year trajectories of long-term benzodiazepine use by adolescents: Patient, provider, and medication factors. Psychiatr. Serv..

[10] McCabe SE, West BT (2014). Medical and nonmedical use of prescription benzodiazepine anxiolytics among U.S. high school seniors. Addict. Behav..

[11] Schepis TS, West BT, Teter CJ, McCabe SE (2016). Prevalence and correlates of co- ingestion of prescription tranquilizers and other psychoactive substances by U.S. high school seniors: Results from a national survey. Addict. Behav..

[12] Piazza PV, Deroche-Gamonet V (2013). A multistep general theory of transition to addiction. Psychopharmacol. (Berl.).

[13] Clark DB, Kirisci L, Tarter RE (1998). Adolescent versus adult onset and the development of substance use disorders in males. Drug Alcohol Depend..

[14] Dawson DA, Goldstein RB, Chou SP, Ruan WJ, Grant BF (2008). Age at first drink and the first incidence of adult-onset DSM-IV alcohol use disorders. Alcohol Clin. Exp. Res.

[15] Luscher C, Ungless MA (2006). The mechanistic classification of addictive drugs. PLoS Med..

[16] Nestler EJ (2001). Molecular basis of long-term plasticity underlying addiction. Nat. Rev. Neurosci..

[17] Tan KR (2010). Neural bases for addictive properties of benzodiazepines. Nature.

[18] Xi ZX, Stein EA (1998). Nucleus accumbens dopamine release modulation by mesolimbic GABAA receptors-an in vivo electrochemical study. Brain Res..

[19] O'Brien DP, White FJ (1987). Inhibition of non-dopamine cells in the ventral tegmental area by benzodiazepines: relationship to A10 dopamine cell activity. Eur. J. Pharmacol..

[20] Tan KR, Rudolph U, Luscher C (2011). Hooked on benzodiazepines: GABAA receptor subtypes and addiction. Trends Neurosci..

[21] Finlay JM, Damsma G, Fibiger HC (1992). Benzodiazepine-induced decreases in extracellular concentrations of dopamine in the nucleus accumbens after acute and repeated administration. Psychopharmacology.

[22] Zetterstrom T, Fillenz M (1990). Local administration of flurazepam has different effects on dopamine release in striatum and nucleus accumbens: A microdialysis study. Neuropharmacology.

[23] Invernizzi R, Pozzi L, Samanin R (1991). Release of dopamine is reduced by diazepam more in the nucleus accumbens than in the caudate nucleus of conscious rats. Neuropharmacology.

[24] Takada K, Murai T, Kanayama T, Koshikawa N (1993). Effects of midazolam and flunitrazepam on the release of dopamine from rat striatum measured by in vivo microdialysis. Br. J. Anaesth..

[25] Gomez AA (2017). Diazepam inhibits electrically evoked and tonic dopamine release in the nucleus accumbens and reverses the effect of amphetamine. ACS Chem. Neurosci..

[26] Schelp SA (2018). Diazepam concurrently increases the frequency and decreases the amplitude of transient dopamine release events in the nucleus accumbens. J. Pharmacol. Exp. Ther..

[27] Chesselet MF (1984). Presynaptic regulation of neurotransmitter release in the brain: Facts and hypothesis. Neuroscience.

[28] Finlay JM, Zigmond MJ, Abercrombie ED (1995). Increased dopamine and norepinephrine release in medial prefrontal cortex induced by acute and chronic stress: Effects of diazepam. Neuroscience.

[29] Brodnik ZD, Batra A, Oleson EB, Espana RA (2019). Local GABA(A) receptor-mediated suppression of dopamine release within the nucleus accumbens. ACS Chem. Neurosci..

[30] Griffiths RR, Weerts EM (1997). Benzodiazepine self-administration in humans and laboratory animals-implications for problems of long-term use and abuse. Psychopharmacology.

[31] Stitzer ML, Griffiths RR, McLellan AT, Grabowski J, Hawthorne JW (1981). Diazepam use among methadone maintenance patients: Patterns and dosages. Drug Alcohol Depend..

[32] Navaratnam V, Foong K (1990). Opiate dependence–the role of benzodiazepines. Curr. Med. Res. Opin..

[33] Walker BM, Ettenberg A (2001). Benzodiazepine modulation of opiate reward. Exp. Clin. Psychopharmacol..

[34] Walker BM, Ettenberg A (2005). Intra-ventral tegmental area heroin-induced place preferences in rats are potentiated by peripherally administered alprazolam. Pharmacol. Biochem. Behav..

[35] Vashchinkina E (2014). Neurosteroid Agonist at GABAA receptor induces persistent neuroplasticity in VTA dopamine neurons. Neuropsychopharmacology.

[36] Iñiguez SD (2010). Extracellular signal-regulated kinase-2 within the ventral tegmental area regulates responses to stress. J. Neurosci..

[37] Ortiz J (1995). Extracellular signal-regulated protein kinases (ERKs) and ERK kinase (MEK) in brain: Regional distribution and regulation by chronic morphine. J. Neurosci..

[38] Russo SJ (2007). IRS2-Akt pathway in midbrain dopamine neurons regulates behavioral and cellular responses to opiates. Nat. Neurosci..

[39] Mumford GK, Evans SM, Fleishaker JC, Griffiths RR (1995). Alprazolam absorption kinetics affects abuse liability. Clin. Pharmacol. Ther..

[40] Reissig CJ, Harrison JA, Carter LP, Griffiths RR (2015). Inhaled vs. oral alprazolam: subjective, behavioral and cognitive effects, and modestly increased abuse potential. Psychopharmacol. (Berl.).

[41] Spear LP (2000). The adolescent brain and age-related behavioral manifestations. Neurosci. Biobehav. Rev..

[42] Andersen SL, Navalta CP (2004). Altering the course of neurodevelopment: A framework for understanding the enduring effects of psychotropic drugs. Int. J. Dev. Neurosci..

[43] Vialou V (2010). DeltaFosB in brain reward circuits mediates resilience to stress and antidepressant responses. Nat. Neurosci..

[44] Warren BL (2013). Neurobiological sequelae of witnessing stressful events in adult mice. Biol. Psych..

[45] Warren BL (2011). Juvenile administration of concomitant methylphenidate and fluoxetine alters behavioral reactivity to reward- and mood-related stimuli and disrupts ventral tegmental area gene expression in adulthood. J. Neurosci..

[46] Iniguez SD (2012). Post-training cocaine exposure facilitates spatial memory consolidation in C57BL/6 mice. Hippocampus.

[47] Bachhuber MA, Hennessy S, Cunningham CO, Starrels JL (2016). Increasing benzodiazepine prescriptions and overdose mortality in the United States, 1996–2013. Am. J. Public Health.

[48] Friedrich JM (2020). Child and adolescent benzodiazepine exposure and overdose in the United States: 16 years of poison center data. Clin. Toxicol. (Phila).

[49] Griebel G (1995). Further evidence that the mouse defense test battery is useful for screening anxiolytic and panicolytic drugs: Effects of acute and chronic treatment with alprazolam. Neuropharmacology.

[50] Cooper SJ (2005). Palatability-dependent appetite and benzodiazepines: New directions from the pharmacology of GABA(A) receptor subtypes. Appetite.

[51] Cooper SJ, Moores WR (1985). Benzodiazepine-induced hyperphagia in the nondeprived rat: Comparisons with CL 218,872, zopiclone, tracazolate and phenobarbital. Pharmacol. Biochem. Behav..

[52] Cooper SJ (1986). Hyperphagic and anorectic effects of beta-carbolines in a palatable food consumption test: Comparisons with triazolam and quazepam. Eur. J. Pharmacol..

[53] Sanger DJ (1984). Chlordiazepoxide-induced hyperphagia in rats: Lack of effect of GABA agonists and antagonists. Psychopharmacology.

[54] Maisonneuve IM, Warner LM, Glick SD (2001). Biphasic dose-related effects of morphine on dopamine release. Drug Alcohol Depend..

[55] Poisnel G, Dhilly M, Le Boisselier R, Barre L, Debruyne D (2009). Comparison of five benzodiazepine-receptor agonists on buprenorphine-induced mu-opioid receptor regulation. J. Pharmacol. Sci..

[56] Randall CK, Kraemer PJ, Dose JM, Carbary TJ, Bardo MT (1992). The biphasic effect of morphine on odor conditioning in neonatal rats. Dev. Psychobiol..

[57] Lerner A, Klein M (2019). Dependence, withdrawal and rebound of CNS drugs: An update and regulatory considerations for new drugs development. Brain Commun..

[58] MacKinnon GL, Parker WA (1982). Benzodiazepine withdrawal syndrome: A literature review and evaluation. Am. J. Drug Alcohol Abuse.

[59] Roy-Byrne PP, Dager SR, Cowley DS, Vitaliano P, Dunner DL (1989). Relapse and rebound following discontinuation of benzodiazepine treatment of panic attacks: Alprazolam versus diazepam. Am. J. Psych..

[60] Busto U, Sellers EM (1991). Pharmacologic aspects of benzodiazepine tolerance and dependence. J. Subst. Abuse Treat..

[61] Kan CC, Hilberink SR, Breteler MH (2004). Determination of the main risk factors for benzodiazepine dependence using a multivariate and multidimensional approach. Compr. Psych..

[62] Schmauss C, Apelt S, Emrich HM (1987). Characterization of benzodiazepine withdrawal in high- and low-dose dependent psychiatric inpatients. Brain Res. Bull..

[63] Moreau JL (1990). Physical dependence induced in DBA/2J mice by benzodiazepine receptor full agonists, but not by the partial agonist Ro 16–6028. Eur. J. Pharmacol..

[64] Dickinson B, Rush PA, Radcliffe AB (1990). Alprazolam use and dependence. A retrospective analysis of 30 cases of withdrawal. West J. Med..

[65] Valerie Curran H (1991). Benzodiazepines, memory and mood: A review. Psychopharmacology.

[66] Engin E (2023). GABAA receptor subtypes and benzodiazepine use, misuse, and abuse. Front. Psych..

[67] Natividad LA, Torres OV, Friedman TC, O’Dell LE (2013). Adolescence is a period of development characterized by short- and long-term vulnerability to the rewarding effects of nicotine and reduced sensitivity to the anorectic effects of this drug. Behav. Brain Res..

[68] McCutcheon JE (2012). Dopamine neurons in the ventral tegmental area fire faster in adolescent rats than in adults. J. Neurophysiol..

[69] Iñiguez SD (2009). Nicotine exposure during adolescence induces a depression-like state in adulthood. Neuropsychopharmacology.

[70] Bolaños CA (2008). Antidepressant treatment can normalize adult behavioral deficits induced by early-life exposure to methylphenidate. Biol. Psych..

[71] Heikkinen AE, Moykkynen TP, Korpi ER (2009). Long-lasting modulation of glutamatergic transmission in VTA dopamine neurons after a single dose of benzodiazepine agonists. Neuropsychopharmacology.

[72] Kelley AE, Berridge KC (2002). The neuroscience of natural rewards: Relevance to addictive drugs. J. Neurosci..

[73] Berhow MT, Hiroi N, Nestler EJ (1996). Regulation of ERK (extracellular signal regulated kinase), part of the neurotrophin signal transduction cascade, in the rat mesolimbic dopamine system by chronic exposure to morphine or cocaine. J. Neurosci..

[74] Krishnan V (2008). AKT signaling within the ventral tegmental area regulates cellular and behavioral responses to stressful stimuli. Biol. Psych..

[75] Lee PS (2003). Insights into the relation between mrna and protein expression patterns: II. Experimental observations in *Escherichia coli*. Biotechnol. Bioeng..

[76] Karve TM, Cheema AK (2011). Small changes huge impact: The role of protein posttranslational modifications in cellular homeostasis and disease. J. Amino Acids.

[77] Di Chiara G (2004). Dopamine and drug addiction: The nucleus accumbens shell connection. Neuropharmacology.

[78] Kim S, Shin JK, Yoon HS, Kim JH (2011). Blockade of ERK phosphorylation in the nucleus accumbens inhibits the expression of cocaine-induced behavioral sensitization in rats. Korean J. Physiol. Pharmacol..

[79] Pierce RC, Wolf ME (2013). Psychostimulant-induced neuroadaptations in nucleus accumbens AMPA receptor transmission. Cold Spring Harb Perspect. Med..

[80] Thomas MJ, Kalivas PW, Shaham Y (2008). Neuroplasticity in the mesolimbic dopamine system and cocaine addiction. Br. J. Pharmacol..

[81] Berridge KC, Robinson TE (2016). Liking, wanting, and the incentive-sensitization theory of addiction. Am. Psychol..

[82] Carlezon WA, Duman RS, Nestler EJ (2005). The many faces of CREB. Trends Neurosci..

[83] Xia Y (2011). Nucleus accumbens medium spiny neurons target non-dopaminergic neurons in the ventral tegmental area. J. Neurosci..

[84] Shibasaki M (2013). Involvement of the K+-Cl- co-transporter KCC2 in the sensitization to morphine-induced hyperlocomotion under chronic treatment with zolpidem in the mesolimbic system. J. Neurochem..

[85] Mehra A, Lee KH, Hatzimanikatis V (2003). Insights into the relation between mRNA and protein expression patterns: I. Theoretical considerations. Biotechnol. Bioeng..

[86] Nestler EJ, Carlezon WA (2006). The mesolimbic dopamine reward circuit in depression. Biol. Psych..

[87] Suzuki S, Chuang TK, Chuang LF, Doi RH, Chuang RY (2001). Morphine upregulates kappa-opioid receptors of human lymphocytes. Adv. Exp. Med. Biol..

[88] Sun A (2015). Decrease of phosphorylated CREB and ERK in nucleus accumbens is associated with the incubation of heroin seeking induced by cues after withdrawal. Neurosci. Lett..

[89] Petursson H (1994). The benzodiazepine withdrawal syndrome. Addiction.

[90] Becker JB, Molenda H, Hummer DL (2001). Gender differences in the behavioral responses to cocaine and amphetamine. Implications for mechanisms mediating gender differences in drug abuse. Ann. N. Y. Acad. Sci..

[91] Agarwal SD, Landon BE (2019). Patterns in outpatient benzodiazepine prescribing in the United States. JAMA Netw. Open.

[92] VanHouten JP, Rudd RA, Ballesteros MF, Mack KA (2019). Drug overdose deaths among women aged 30–64 years—United States, 1999–2017. MMWR Morb. Mortal. Wkly. Rep..

