# Supplementary material for: Alprazolam exposure during adolescence induces long-lasting dysregulation in reward sensitivity to morphine and second messenger signaling in the VTA-NAc pathway
Source: Sci Rep. 2023 Jul 5;13:10872. doi: 10.1038/s41598-023-37696-8 (PMC10322866; doi:10.1038/s41598-023-37696-8)
Supplement: Supplementary file 1 — Supplementary Figures. [file 41598_2023_37696_MOESM1_ESM.docx]

**Figure S1.** Total ERK1/2-related protein expression, within the VTA after chronic ALP exposure (short-term; 24 h after the last injection). Blots correspond to Figures 4*B* and 4*C* where total ERK1, ERK2, and GAPDH are represented.


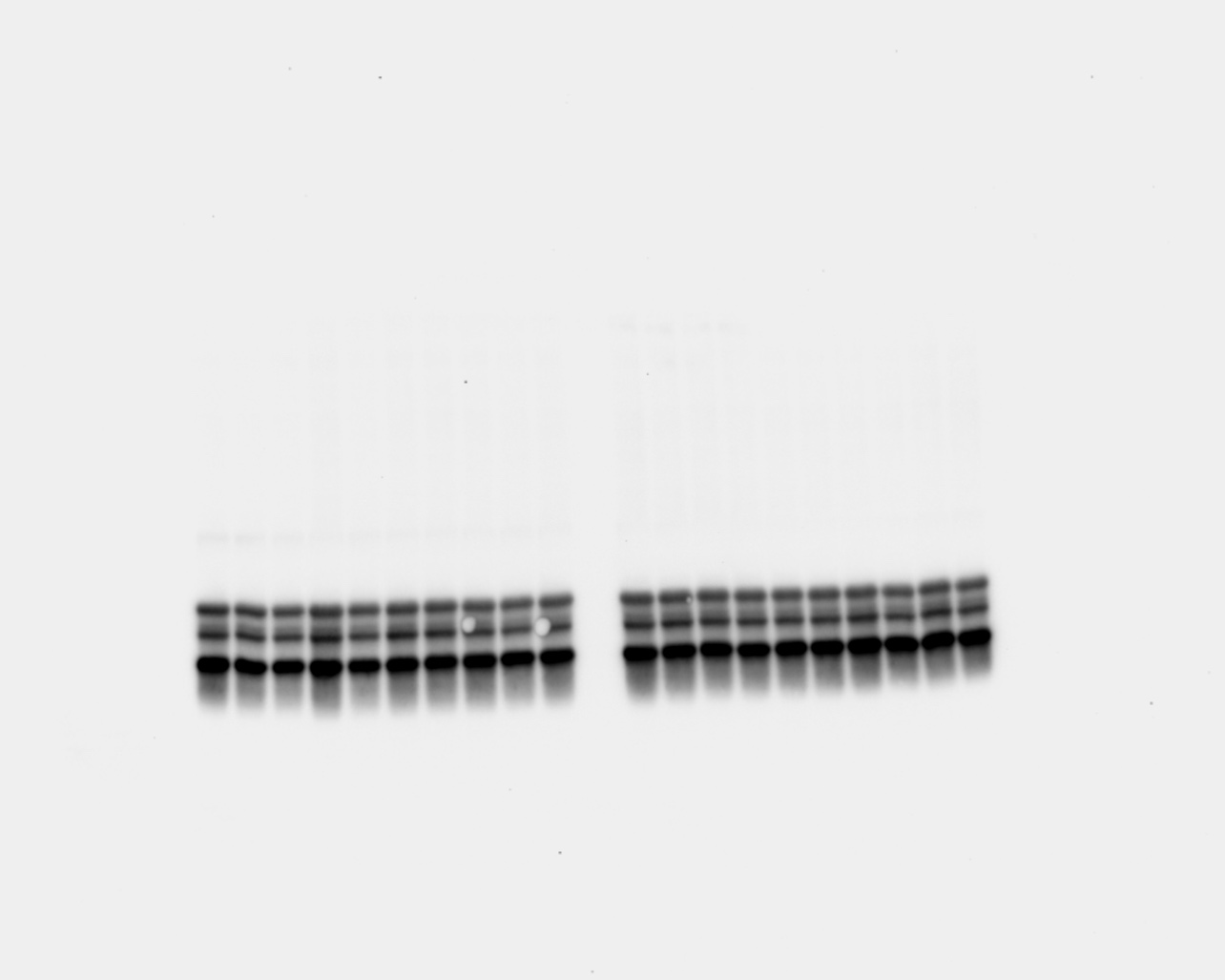


44 kDA T-ERK1

42 kDA T-ERK2

37 kDA GAPDH

VEH ALP VEH ALP VEH

**Figure S2.** Total ERK1/2-related protein expression, within the VTA after chronic ALP exposure (short-term; 24 h after the last injection). Blots correspond to Figures 4*D* and 4*E* where total CREB and AKT are represented.


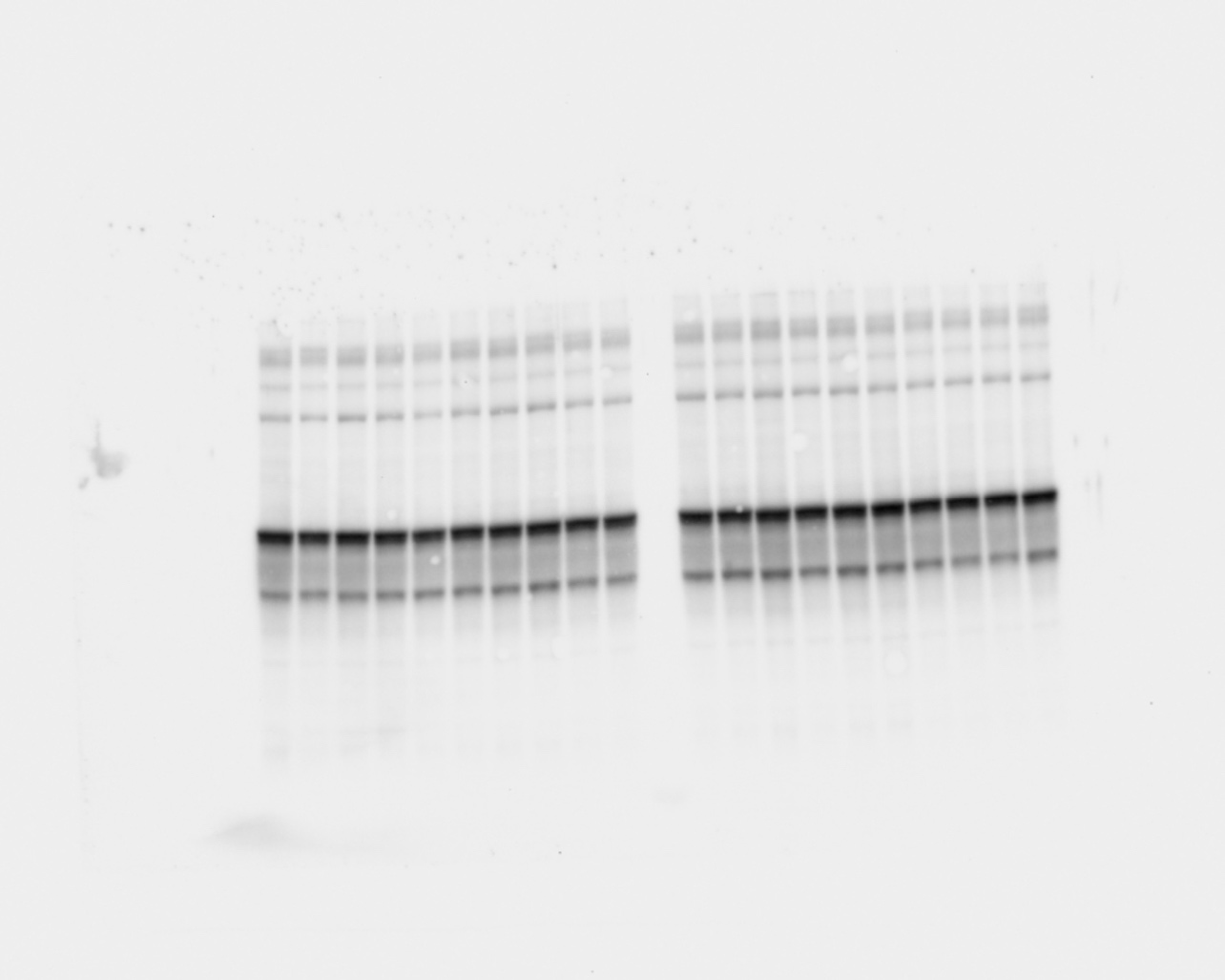


60 kDA T-AKT

43 kDA T-CREB

VEH ALP VEH ALP VEH

**Figure S3.** ERK1/2-related protein phosphorylation, within the VTA after chronic ALP exposure (short-term; 24 h after the last injection). Blots correspond to Figures 4*B* and 4*C* where phosphorylated ERK1 and ERK2 are represented.


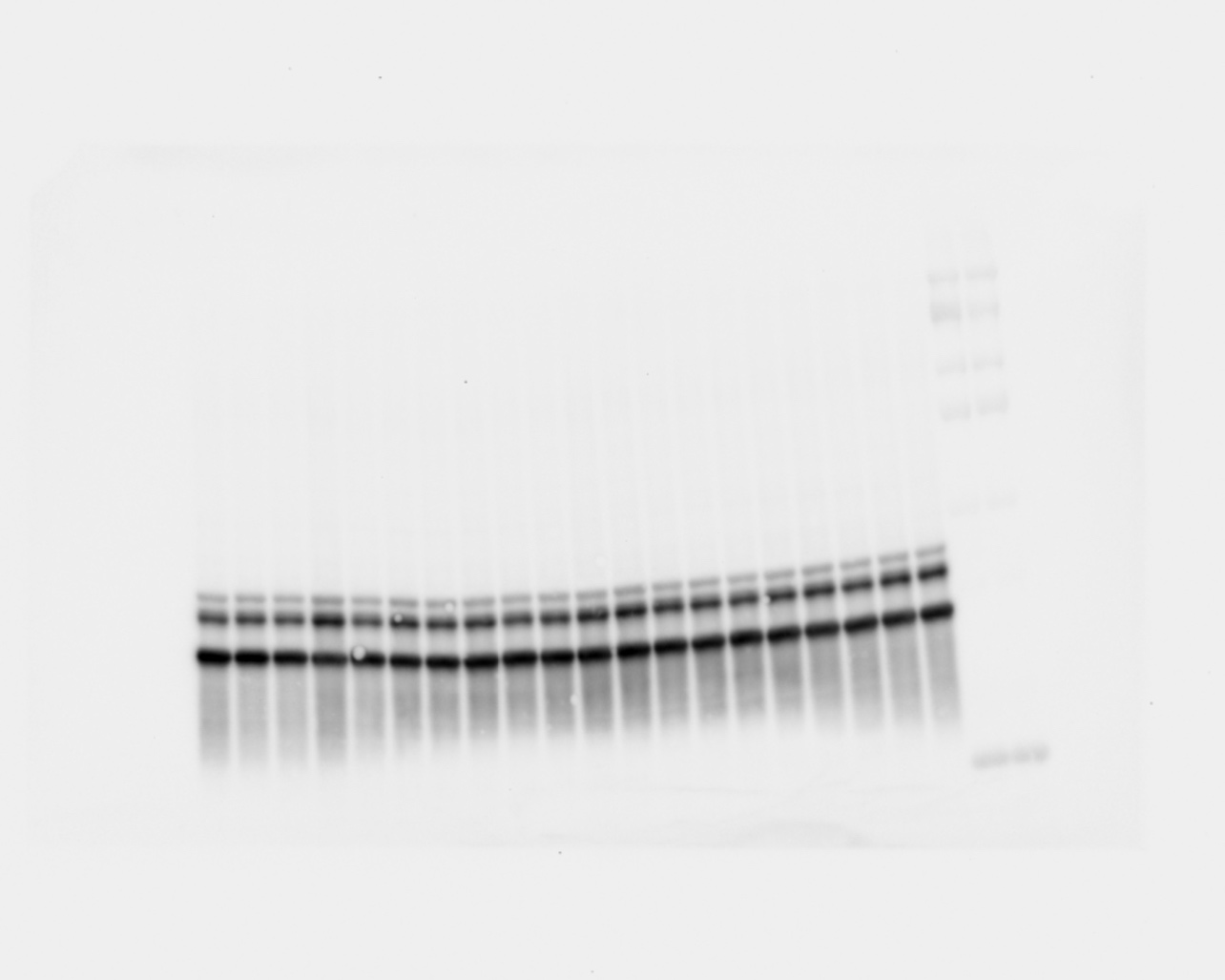


44 kDA P-ERK1

42 kDA P-ERK2

37 kDA GAPDH

VEH ALP VEH ALP VEH

**Figure S4.** ERK1/2-related protein phosphorylation, within the VTA after chronic ALP exposure (short-term; 24 h after the last injection). Blots correspond to Figures 4*D* and 4*E* where phosphorylated CREB and AKT are represented.


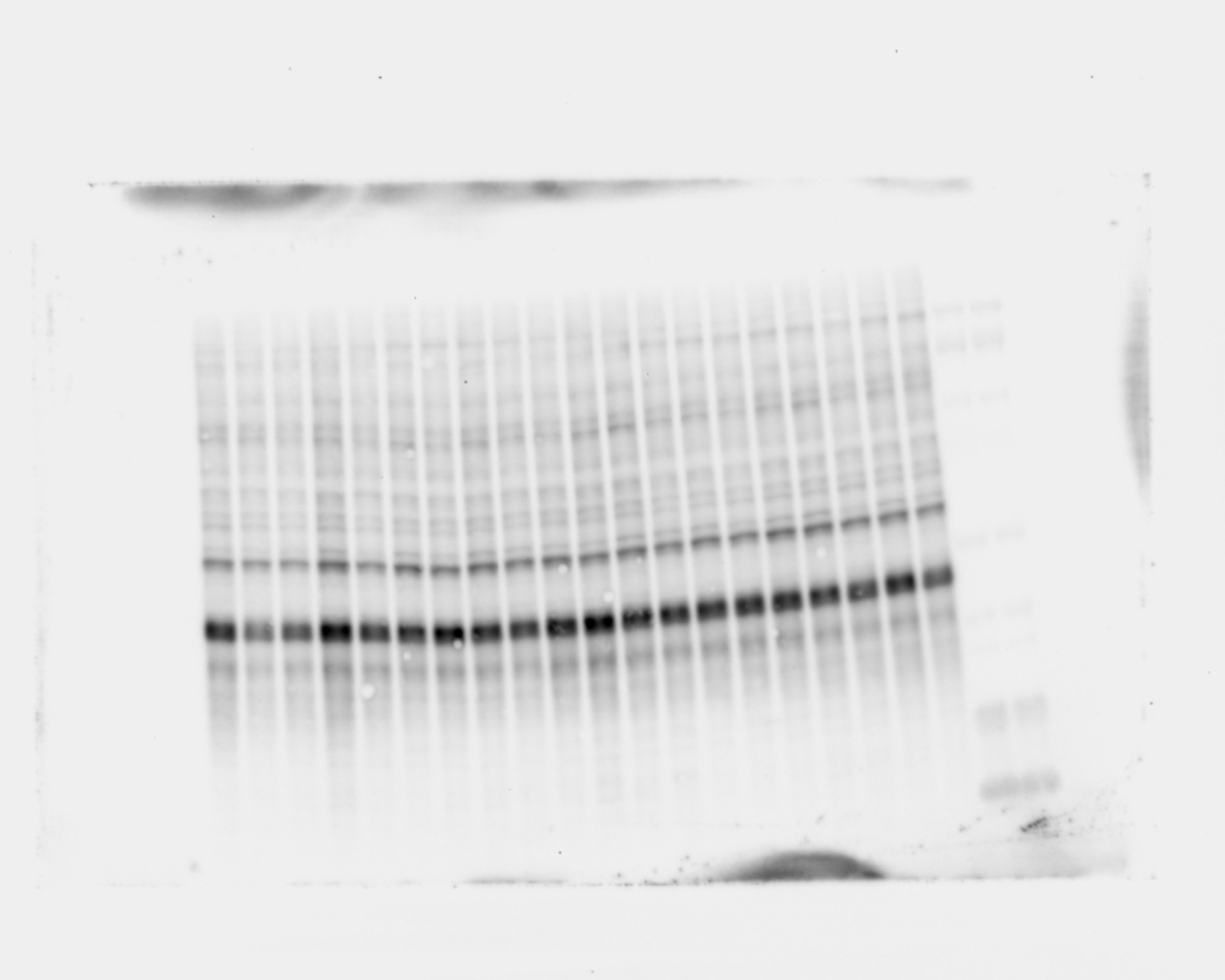


60 kDA P-AKT

43 kDA P-CREB

VEH ALP VEH ALP VEH

**Figure S5.** Total ERK1/2-related protein expression, within the NAc after chronic ALP exposure (short-term; 24 h after the last injection). Blots correspond to Figures 6*B* and 6*C* where total ERK1 and ERK2 are represented.


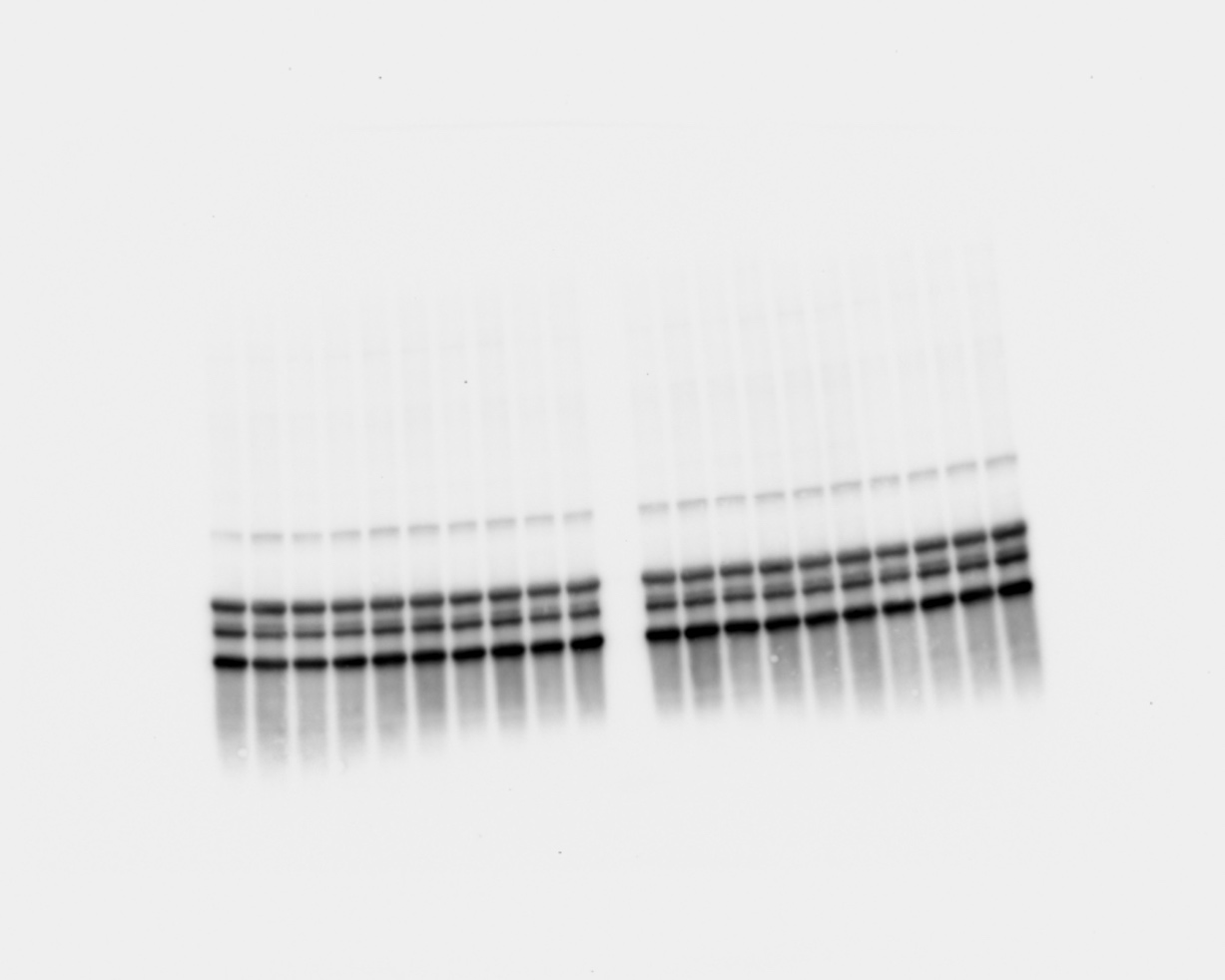


44 kDA T-ERK1

42 kDA T-ERK2

37 kDA GAPDH

VEH ALP VEH ALP VEH

**Figure S6.** Total ERK1/2-related protein expression, within the NAc after chronic ALP exposure (short-term; 24 h after the last injection). Blots correspond to Figures 6*D* and 6*E* where total were total CREB and AKT are represented.


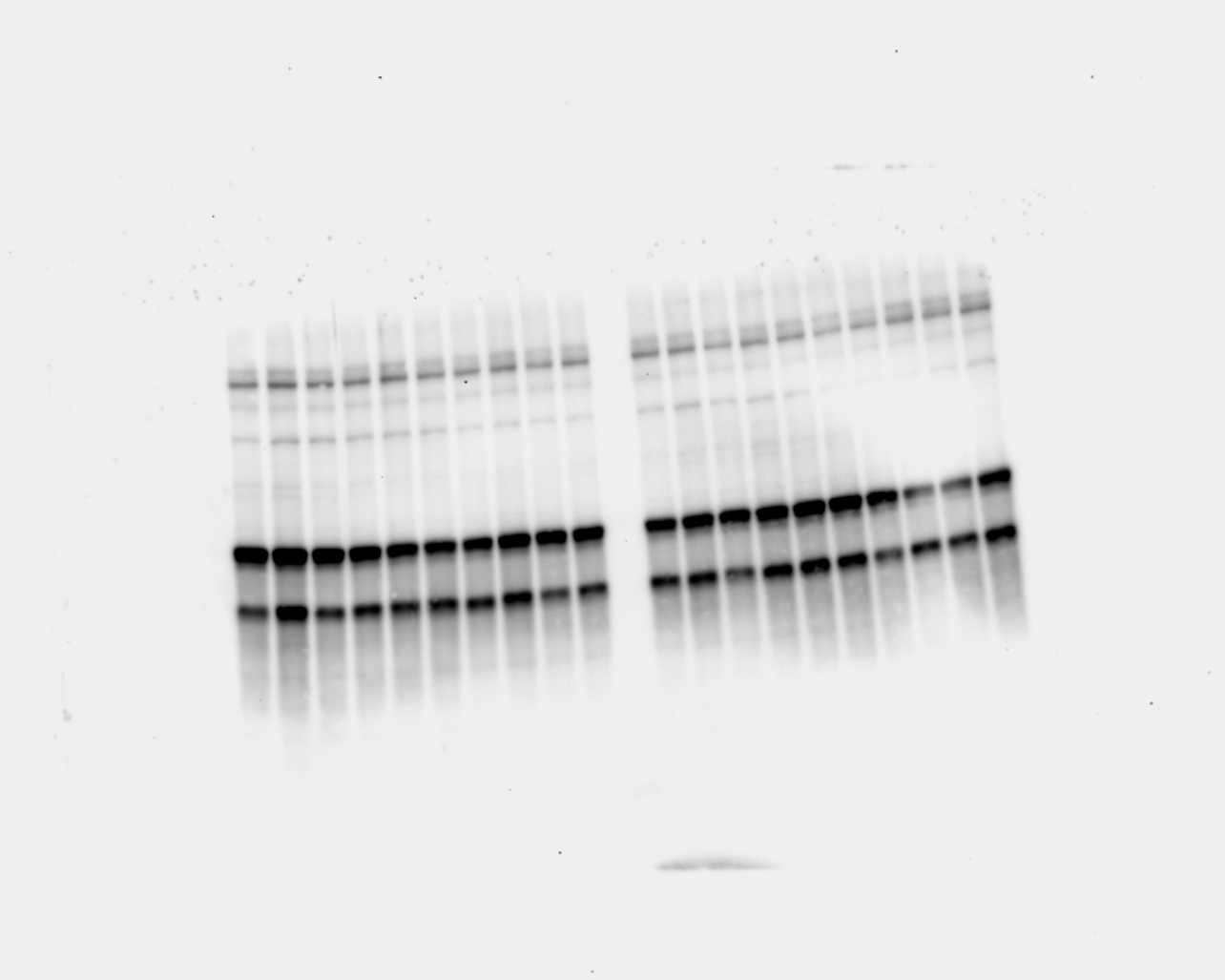


60 kDA T-AKT

43 kDA T-CREB

VEH ALP VEH ALP VEH

**Figure S7.** ERK1/2-related protein phosphorylation, within the NAc after chronic ALP exposure (short-term; 24 h after the last injection). Blots correspond to Figures 6*B* and 6*C* where phosphorylated ERK1, ERK2, and GAPDH are represented.


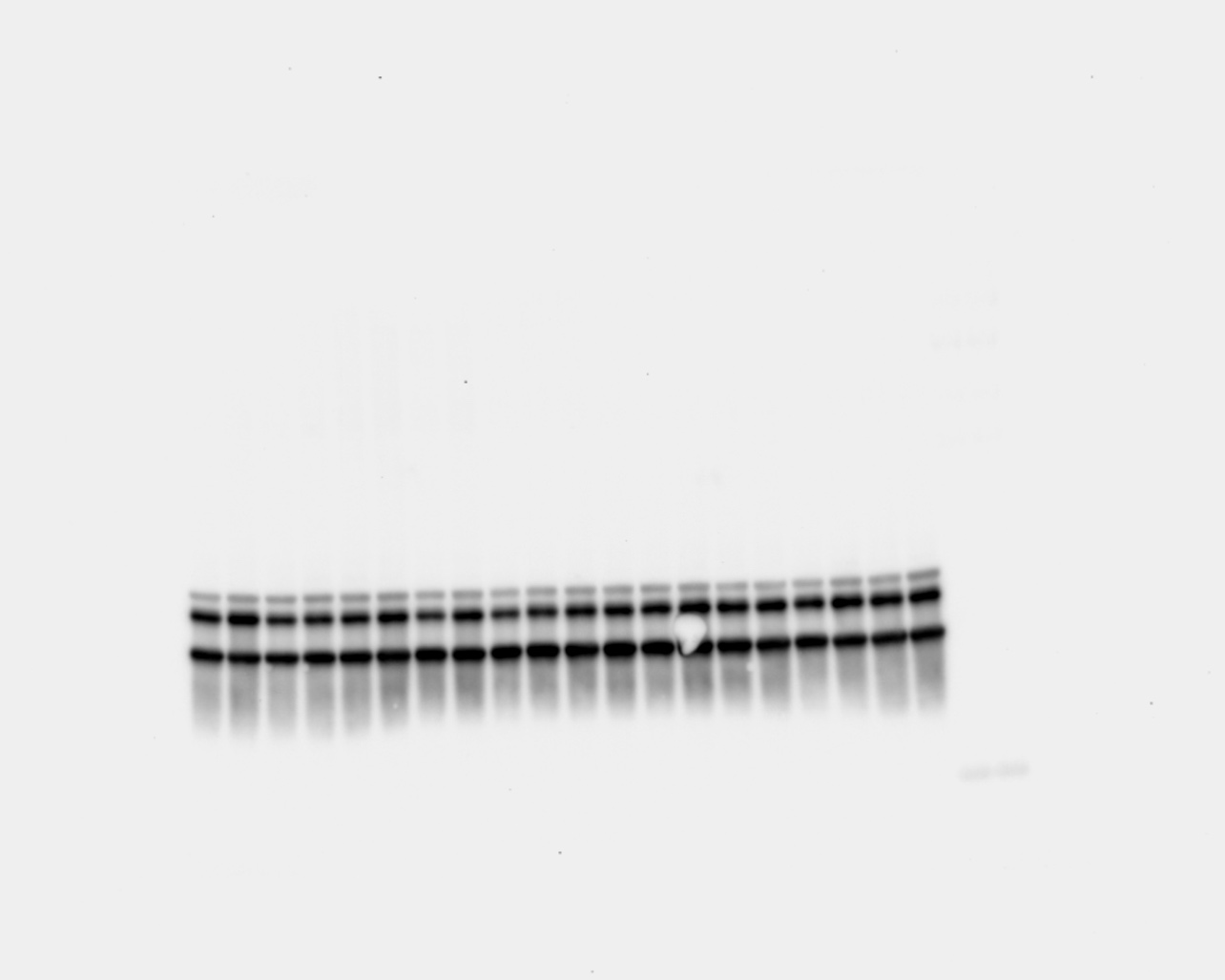


44 kDA P-ERK1

42 kDA P-ERK2

37 kDA GAPDH

VEH ALP VEH ALP VEH

**Figure S8.** ERK1/2-related protein phosphorylation, within the NAc after chronic ALP exposure (short-term; 24 h after the last injection). Blots correspond to Figure 6*D* where phosphorylated CREB is represented.


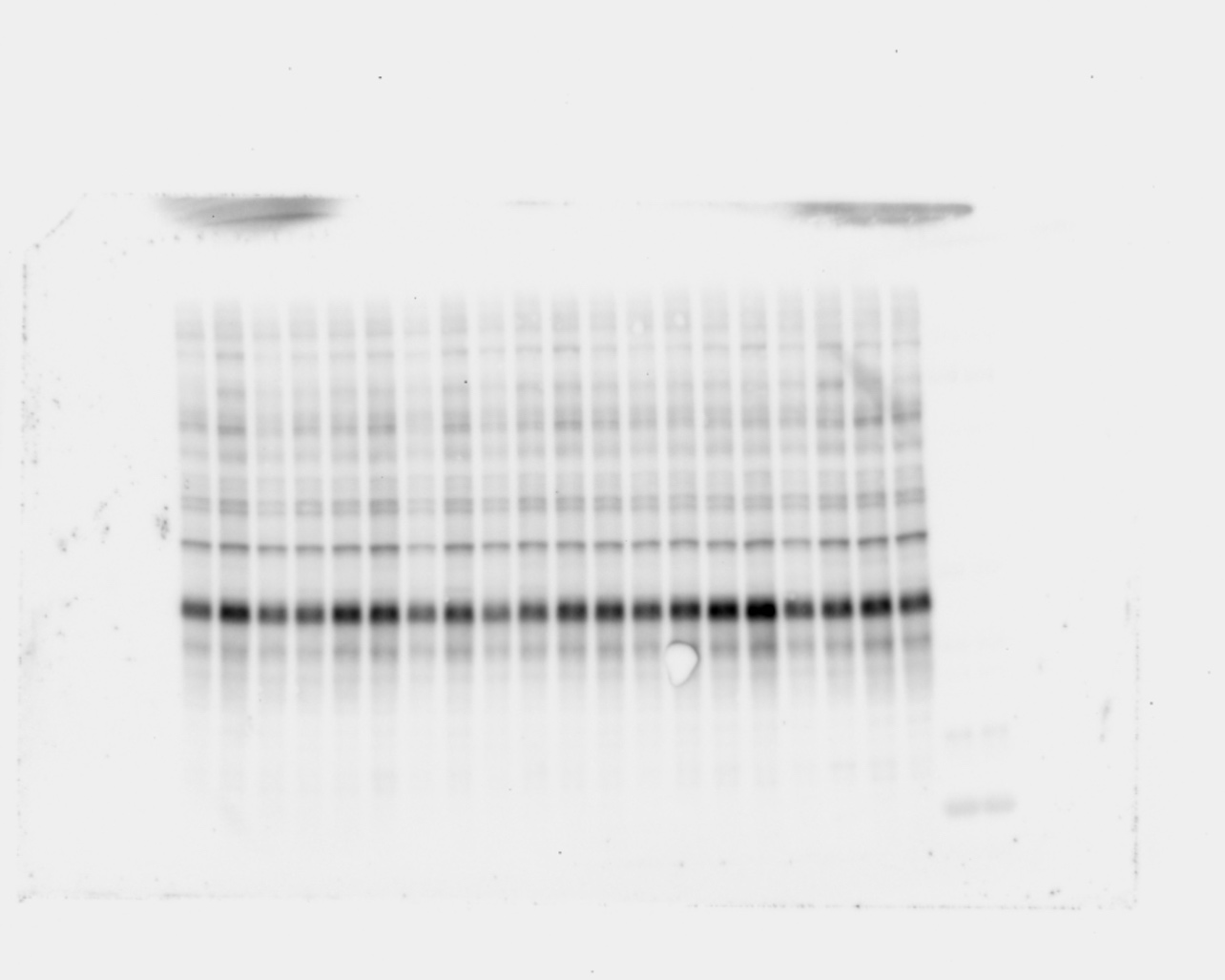


43 kDA P-CREB

VEH ALP VEH ALP VEH

**Figure S9.** ERK1/2-related protein phosphorylation, within the NAc after chronic ALP exposure (short-term; 24 h after the last injection). Blots correspond to Figures 6*E* where phosphorylated AKT is represented.


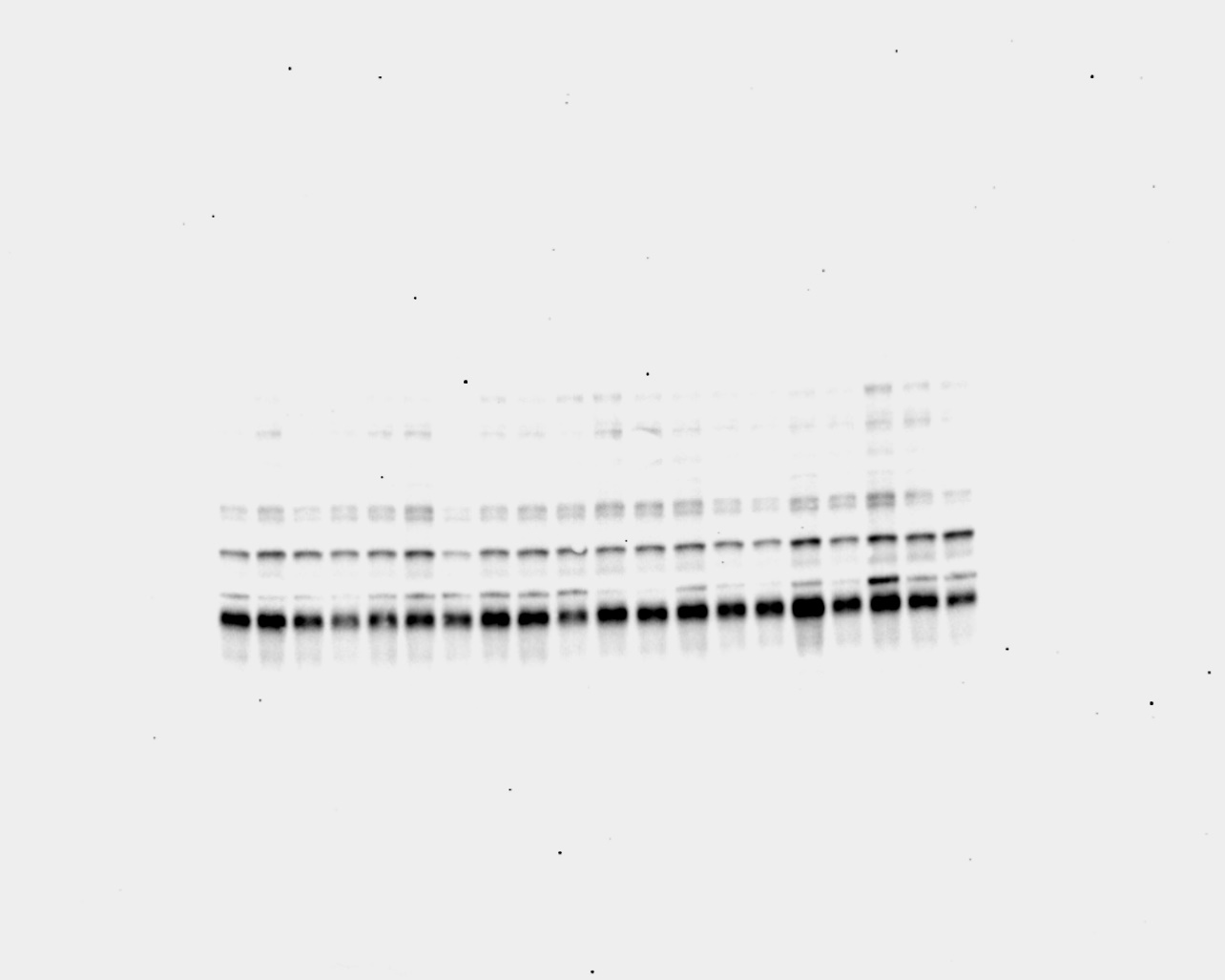


43 kDA P-AKT

VEH ALP VEH ALP VEH

**Figure S10.** Total ERK1/2-related protein expression, within the VTA after chronic ALP exposure (long-term; 1 month after the last injection). Blots correspond to Figures 8*B* and 8*C* where total ERK1 and ER2 are represented.


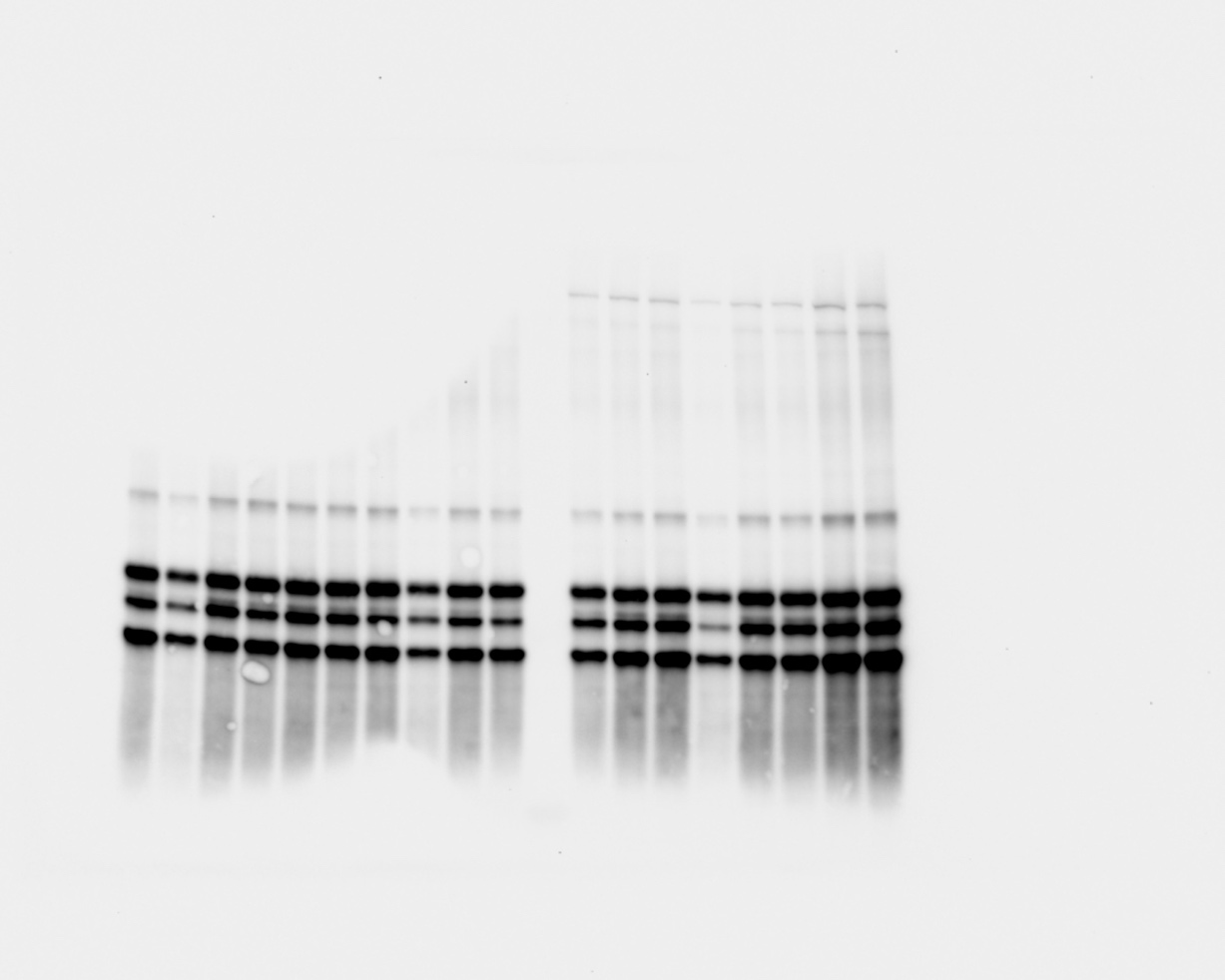


44 kDA T-ERK1

42 kDA T-ERK2

37 kDA GAPDH

VEH ALP VEH ALP VEH

**Figure S11.** Total ERK1/2-related protein expression, within the VTA after chronic ALP exposure (long-term; 1 month after the last injection). Blots correspond to Figures 8*D* and 8*E* where total CREB and AKT are represented.


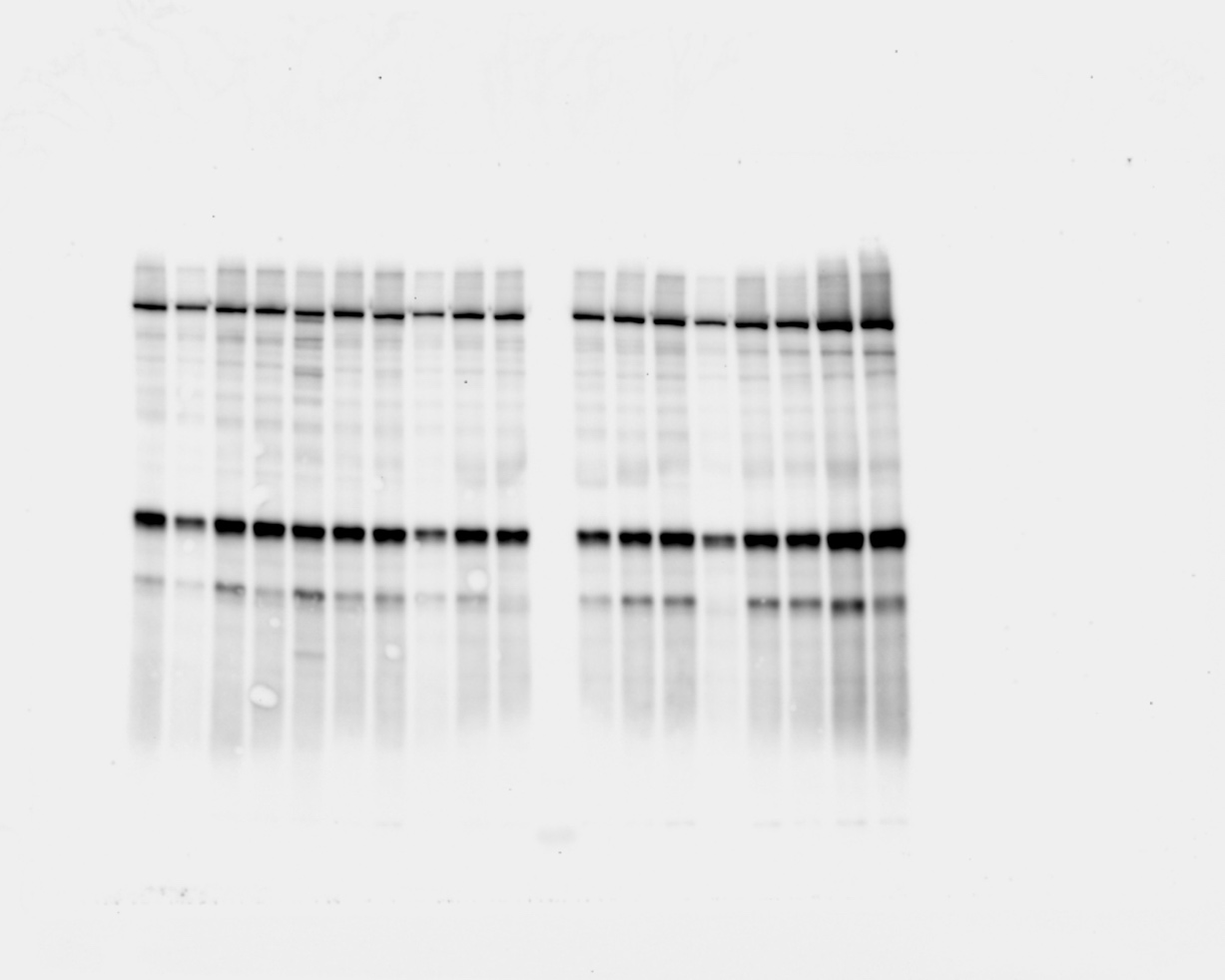


60 kDA T-AKT

43 kDA T-CREB

VEH ALP VEH ALP VEH

**Figure S12.** ERK1/2-related protein phosphorylation within the VTA after chronic ALP exposure (long-term; 1 month after the last injection). Blots correspond to Figures 8*B* and 8*C* where phosphorylated ERK1, ERK2, and GAPDH are represented.


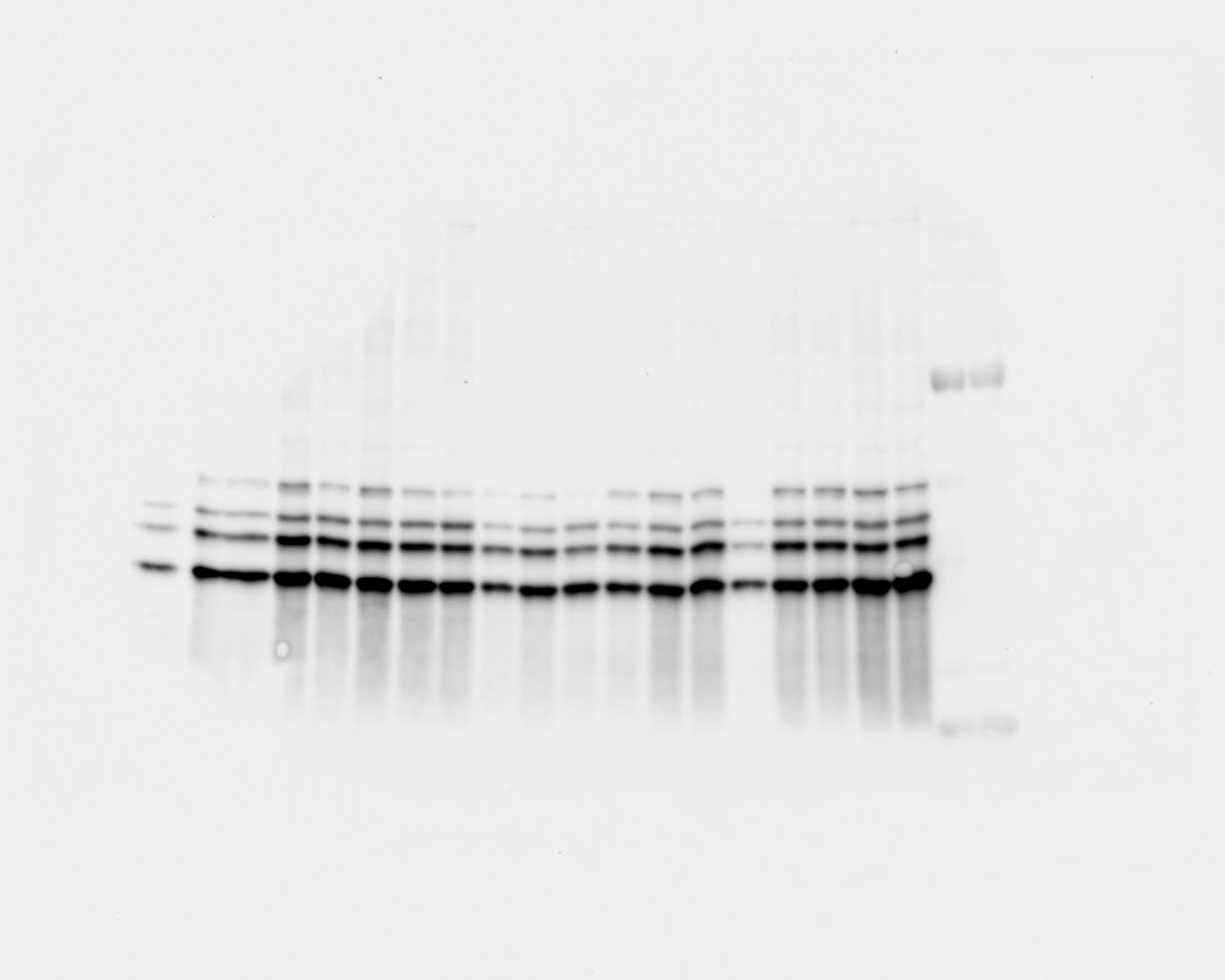


44 kDA P-ERK1

42 kDA P-ERK2

37 kDA GAPDH

VEH ALP VEH ALP VEH

**Figure S13.** ERK1/2-related protein phosphorylation within the VTA after chronic ALP exposure (long-term; 1 month after the last injection). Blots correspond to Figures 8*D* and 8*E* where phosphorylated CREB and AKT are represented.


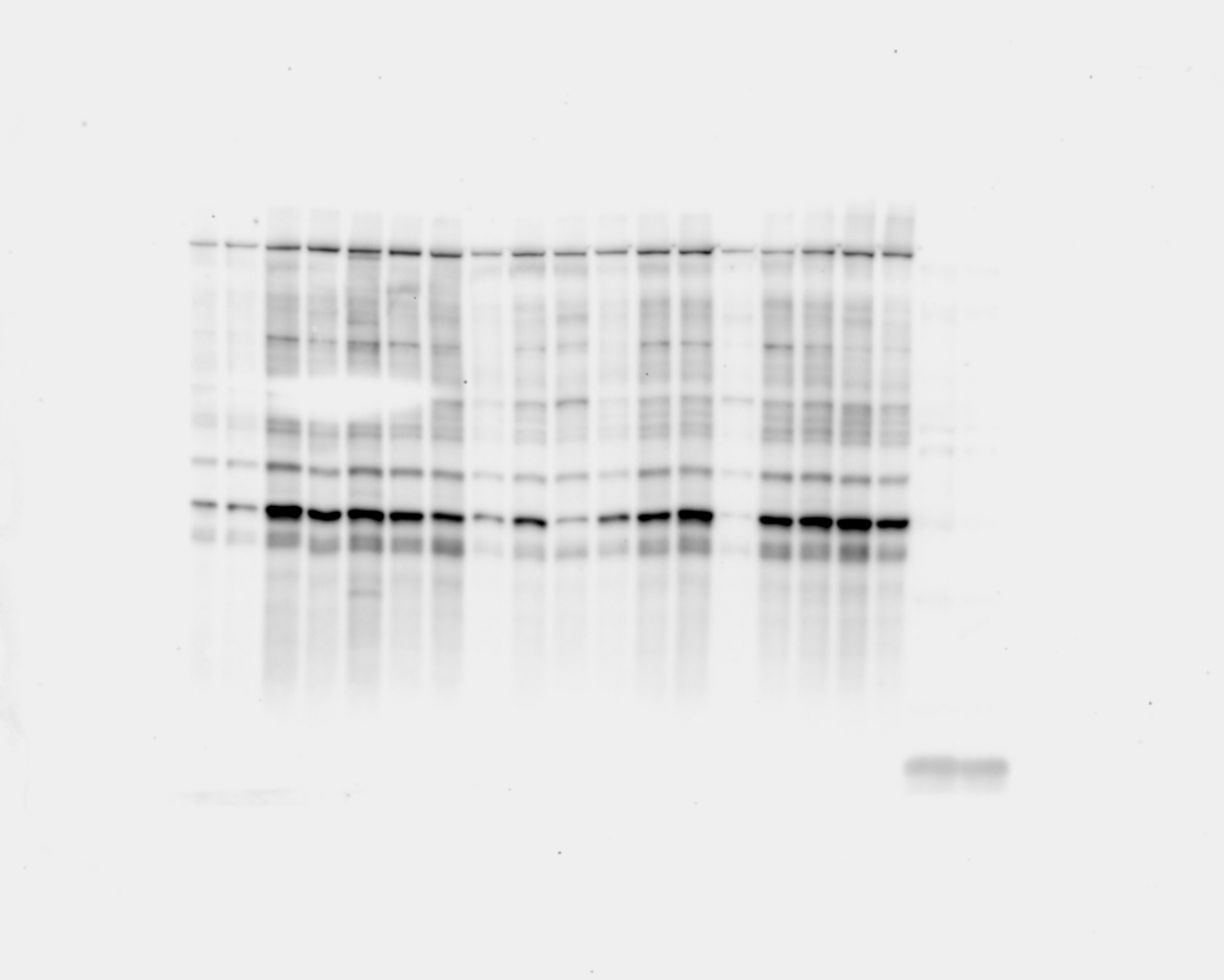


60 kDA P-AKT

43 kDA P-CREB

VEH ALP VEH ALP VEH

**Figure S14.** Total ERK1/2-related protein expression, within the NAc after chronic ALP exposure (long-term; 1 month after the last injection). Blots correspond to Figures 10*B* and 10*C* where total ERK1 and ERK2 are represented.


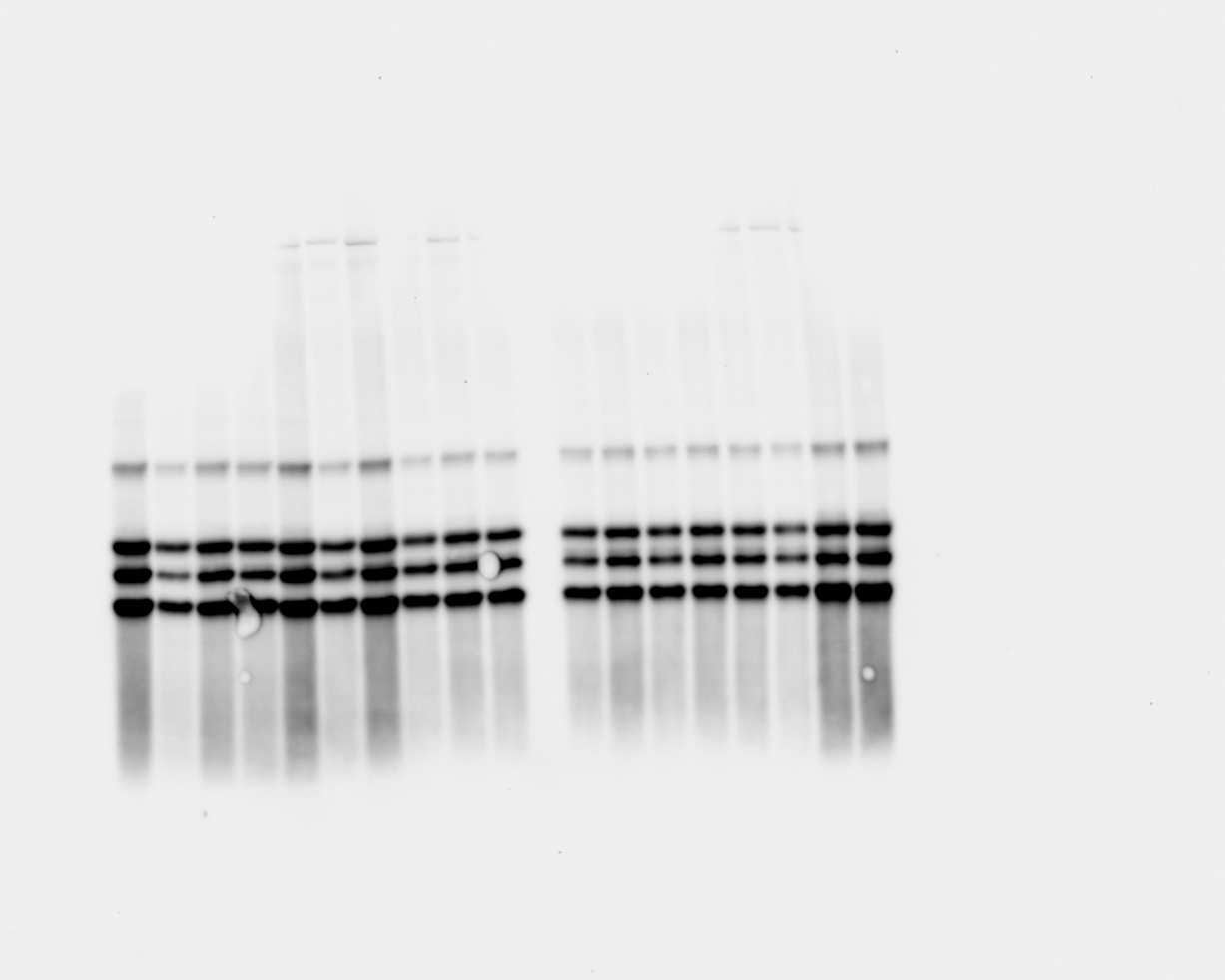


44 kDA T-ERK1

42 kDA T-ERK2

37 kDA GAPDH

VEH ALP VEH ALP VEH

**Figure S15.** Total ERK1/2-related protein expression, within the NAc after chronic ALP exposure (long-term; 1 month after the last injection). Blots correspond to Figures 10*D* and 10*E* where total CREB and AKT are represented.


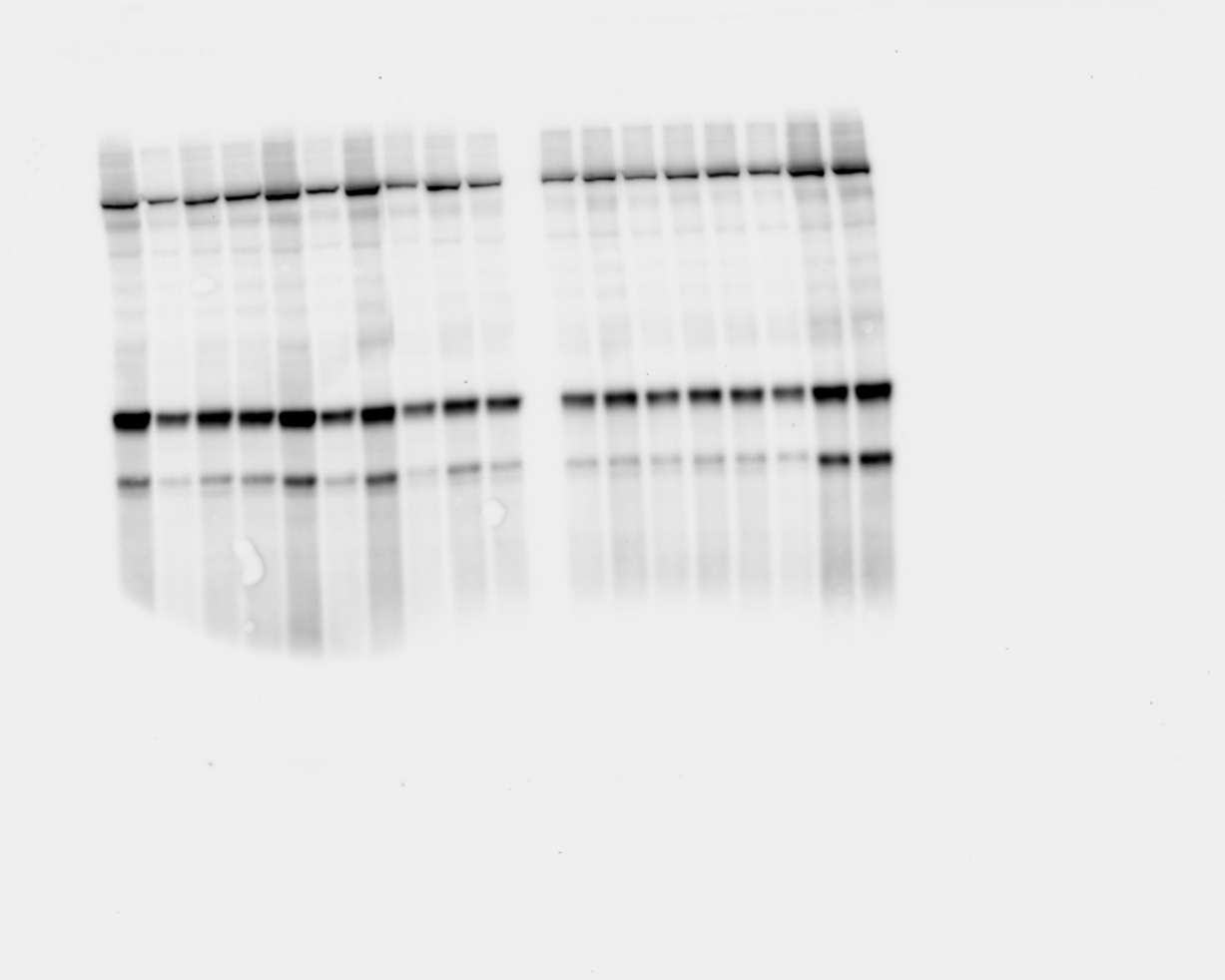


60 kDA T-AKT

43 kDA T-CREB

VEH ALP VEH ALP VEH

**Figure S16.** ERK1/2-related protein phosphorylation within the NAc after chronic ALP exposure (long-term; 1 month after the last injection). Blots correspond to Figures 10*B* and 10*C* where phosphorylated ERK1, ERK2, and GAPDH are represented.


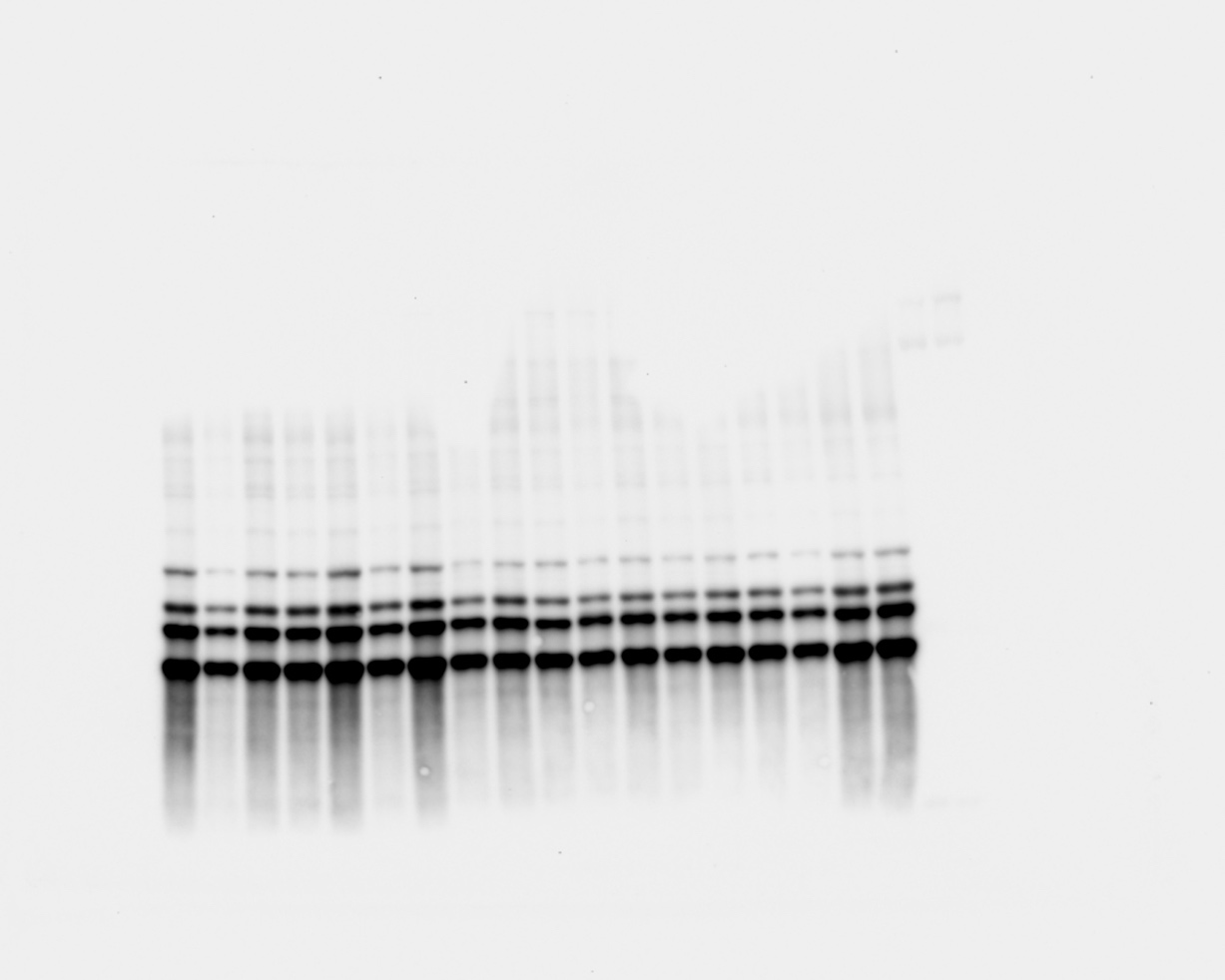


44 kDA P-ERK1

42 kDA P-ERK2

37 kDA GAPDH

VEH ALP VEH ALP VEH

**Figure S17.** ERK1/2-related protein phosphorylation within the NAc after chronic ALP exposure (long-term; 1 month after the last injection). Blots correspond to Figures 10*D* and 10*E* where phosphorylated CREB and AKT are represented.


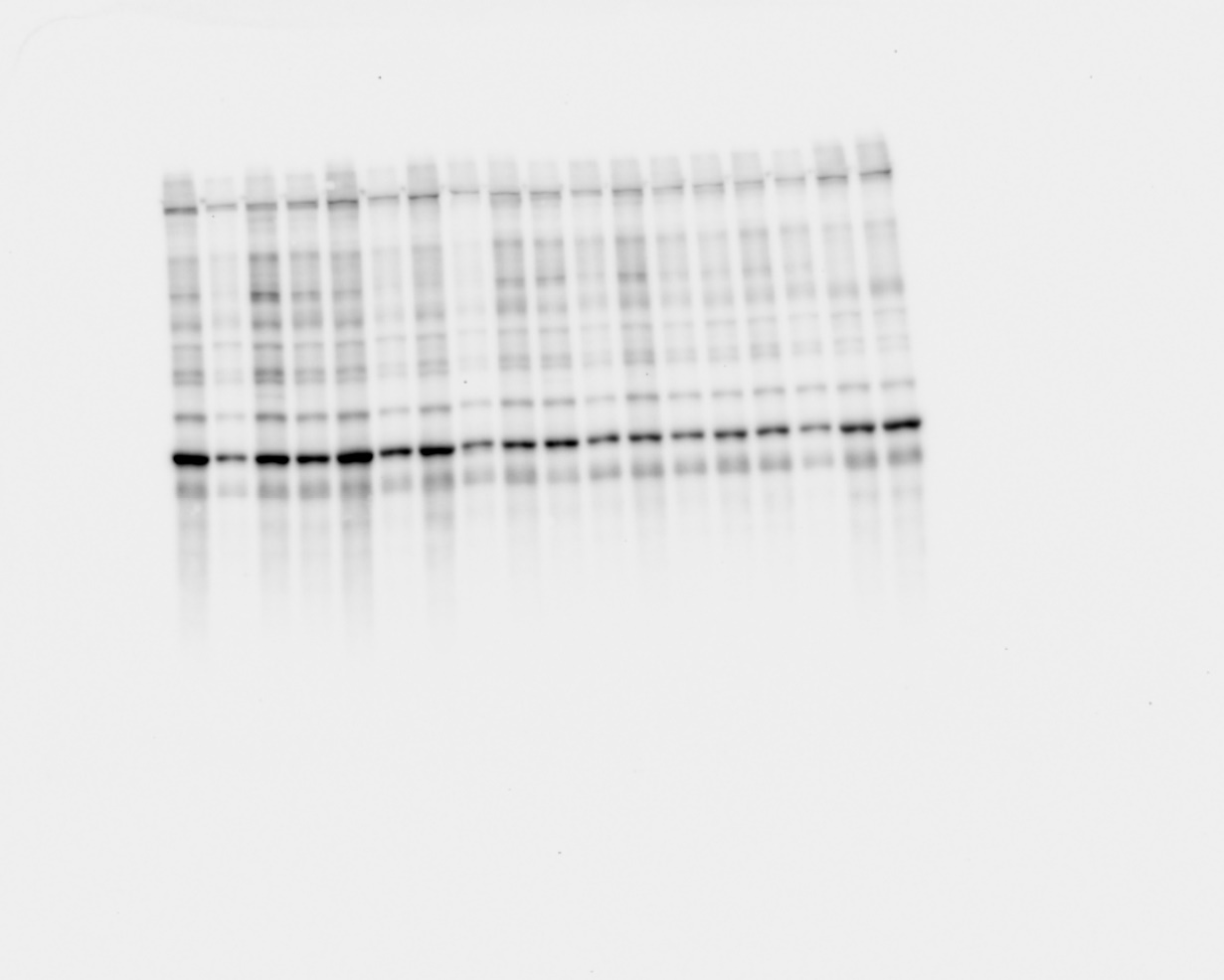


60 kDA P-AKT

43 kDA P-CREB

VEH ALP VEH ALP VEH
