# Supplementary material for: Alprazolam exposure during adolescence induces long-lasting dysregulation in reward sensitivity to morphine and second messenger signaling in the VTA-NAc pathway
Source: Sci Rep. 2023 Jul 5;13:10872. doi: 10.1038/s41598-023-37696-8 (PMC10322866; doi:10.1038/s41598-023-37696-8)

Supplemental Figures (SREP-22-03371A)

**Figure 1.** Effects of repeated alprazolam (ALP) administration during adolescence in total protein expression of ERK-related genes within the VTA 24-hr after the last injection.


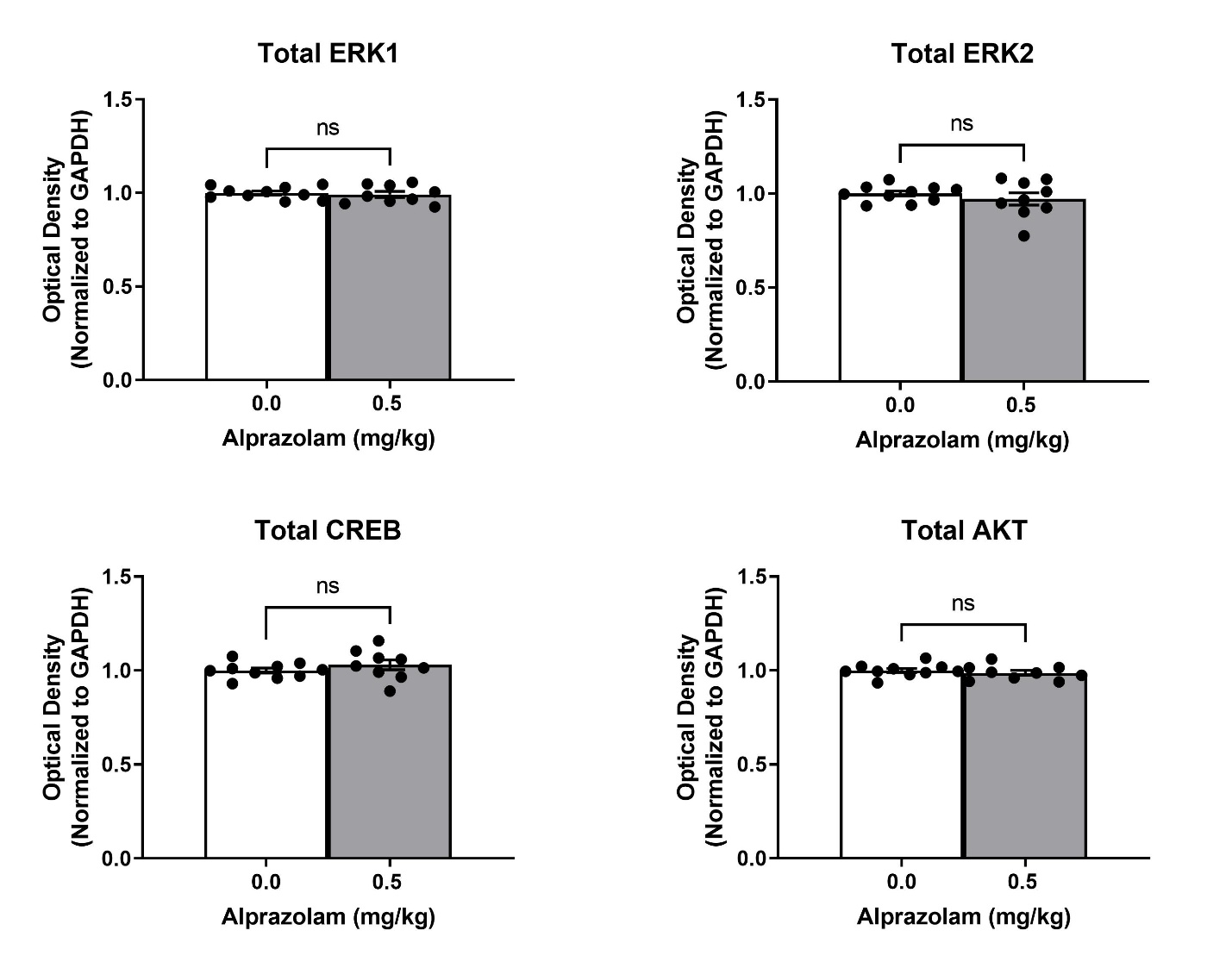


**Figure 2.** Effects of repeated alprazolam (ALP) administration during adolescence in total protein expression of ERK-related genes within the NAc 24-hr after the last injection.


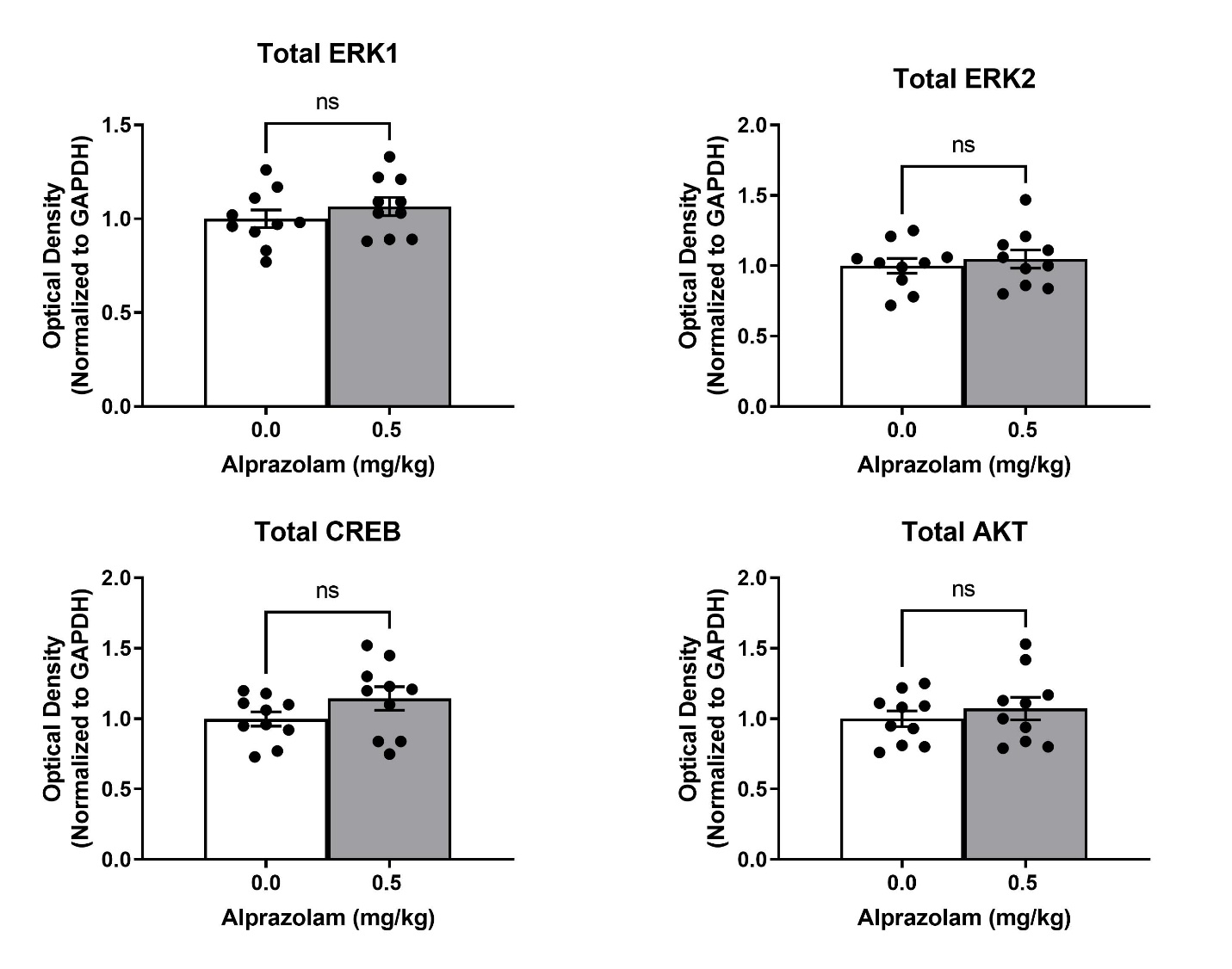


**Figure 3.** Effects of repeated alprazolam (ALP) administration during adolescence in total protein expression of ERK-related genes within the VTA 1-month after the last injection.


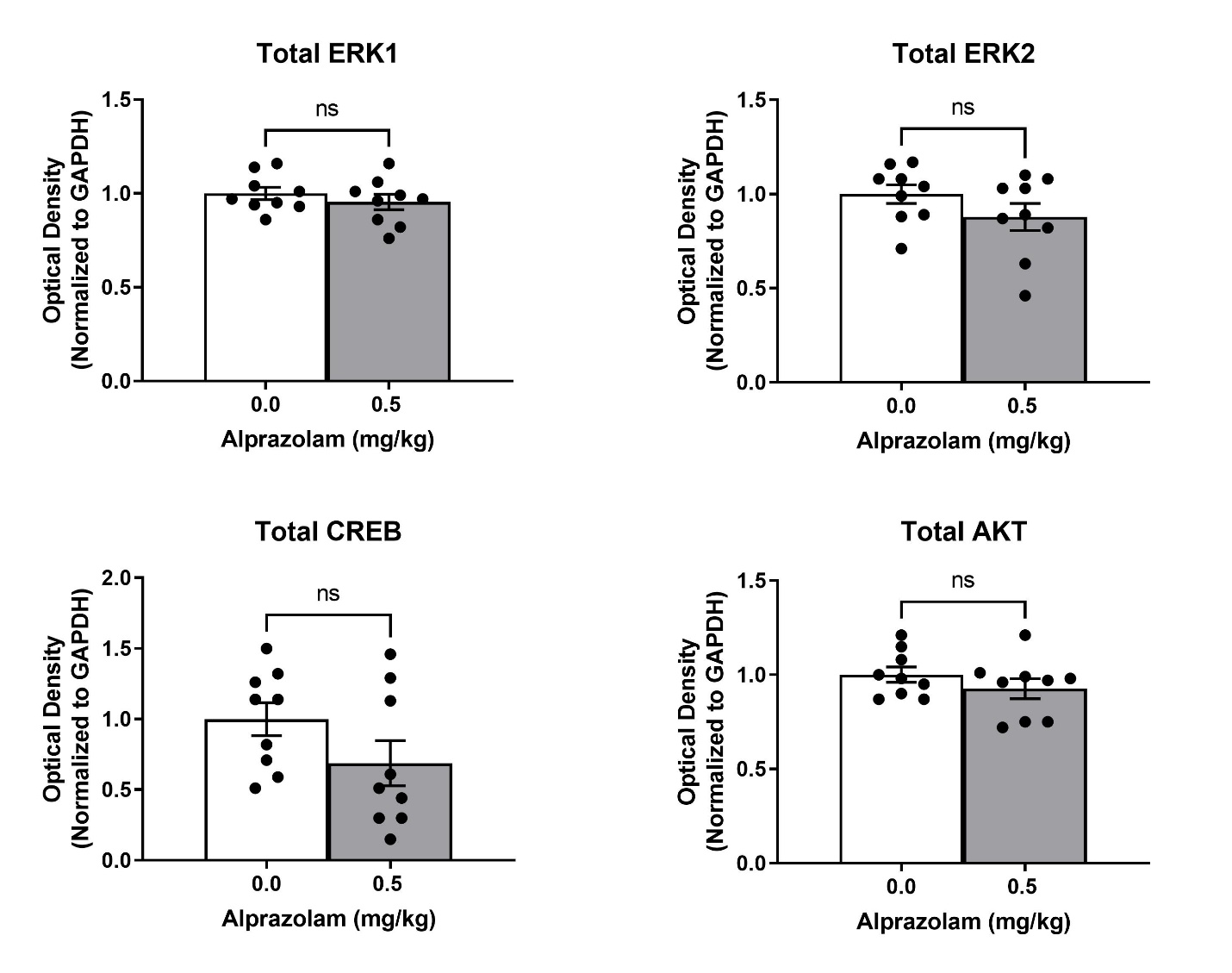


**Figure 4.** Effects of repeated alprazolam (ALP) administration during adolescence in total protein expression of ERK-related genes within the NAc 1-month after the last injection.


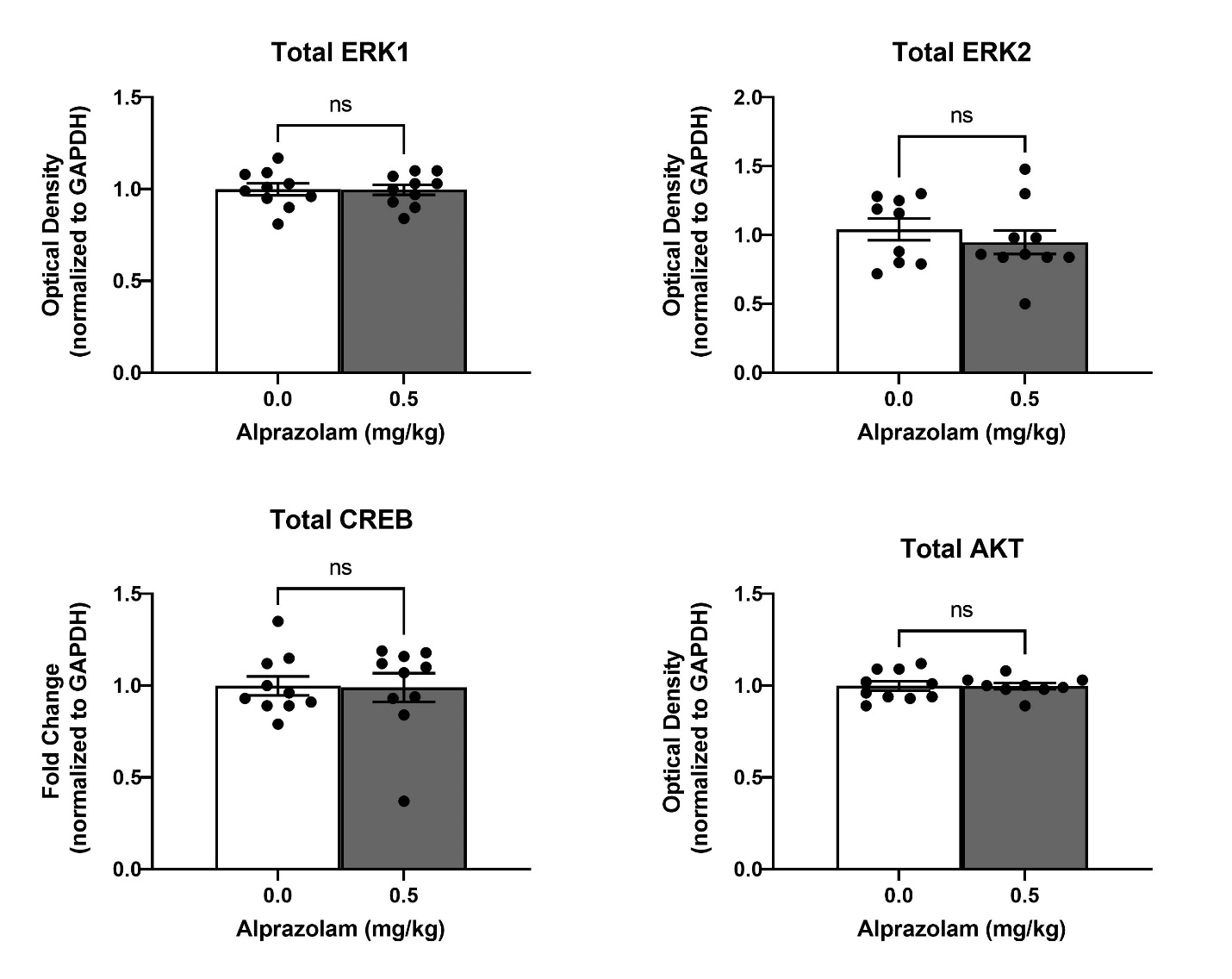

Supplement: Supplementary file 2 — Supplementary Figures. [file 41598_2023_37696_MOESM2_ESM.doc]
